# LP-184, a Novel Acylfulvene Molecule, Exhibits Anticancer Activity against Diverse Solid Tumors with Homologous Recombination Deficiency

**DOI:** 10.1158/2767-9764.CRC-23-0554

**Published:** 2024-05-06

**Authors:** Aditya Kulkarni, Jianli Zhou, Neha Biyani, Umesh Kathad, Partha P. Banerjee, Shiv Srivastava, Zsombor Prucsi, Kamil Solarczyk, Kishor Bhatia, Reginald B. Ewesuedo, Panna Sharma

**Affiliations:** 1 Lantern Pharma Inc., Dallas, Texas.; 2 Department of Biochemistry, Molecular and Cellular Biology, Georgetown University Medical Center, Washington, District of Columbia.; 3 intoDNA S.A. Kraków, Poland.

## Abstract

Homologous recombination (HR)-related gene alterations are present in a significant subset of prostate, breast, ovarian, pancreatic, lung, and colon cancers rendering these tumors as potential responders to specific DNA damaging agents. A small molecule acylfulvene prodrug, LP-184, metabolizes to an active compound by the oxidoreductase activity of enzyme prostaglandin reductase 1 (PTGR1), which is frequently elevated in multiple solid tumor types. Prior work demonstrated that cancer cell lines deficient in a spectrum of DNA damage repair (DDR) pathway genes show increased susceptibility to LP-184. Here, we investigated the potential of LP-184 in targeting multiple tumors with impaired HR function and its mechanism of action as a DNA damaging agent. LP-184 induced elevated DNA double-strand breaks in HR deficient (HRD) cancer cells. Depletion of key HR components BRCA2 or ataxia telangiectasia mutated (ATM) in cancer cells conferred up to 12-fold increased sensitivity to the LP-184. LP-184 showed nanomolar potency in a diverse range of HRD cancer models, including prostate cancer organoids, leiomyosarcoma cell lines, and patient-derived tumor graft models of lung, pancreatic, and prostate cancers. LP-184 demonstrated complete, durable tumor regression in 10 patient-derived xenograft (PDX) models of HRD triple-negative breast cancer (TNBC) including those resistant to PARP inhibitors (PARPi). LP-184 further displayed strong synergy with PARPi in ovarian and prostate cancer cell lines as well as in TNBC PDX models. These preclinical findings illustrate the potential of LP-184 as a pan-HRD cancer therapeutic. Taken together, our results support continued clinical evaluation of LP-184 in a large subset of HRD solid tumors.

**Significance:**

New agents with activity against DDR-deficient solid tumors refractory to standard-of-care therapies are needed. We report multiple findings supporting the potential for LP-184, a novel alkylating agent with three FDA orphan drug designations, to fill this void clinically: strong nanomolar potency; sustained, durable regression of solid tumor xenografts; synthetic lethality with HR defects. LP-184 adult phase IA trial to assess safety in advanced solid tumors is ongoing.

## Introduction

Homologous recombination (HR) is a type of DNA damage repair (DDR) pathway which plays a central role in defending and maintaining genomic stability by repairing DNA double-strand breaks (DSB; ref. 1). Disruption in HR pathways leads to homologous recombination deficiency (HRD) associated with accumulation of increasing levels of DNA mutations in the absence of an intact DSB repair system (2). Advancements in genetic testing have predicted the prevalence of HRD in approximately 17.4% of tumors spanning across 21 different cancer types (3). Prostate, breast, pancreatic, ovarian, lung cancers, and rare cancer types such as leiomyosarcomas (LMS) frequently harbor HRD (2, 4). Tumors carrying HRD become dependent on alternative repair strategies making them vulnerable to targeted agents offering a biomarker-driven approach for clinical development.

PARP inhibitors (PARPi) are a class of such targeted agents that exploit the HRD observed in cancer cells with BRCA mutations. By blocking PARP enzyme activity, these inhibitors impede the repair of single-strand breaks (SSB) and promote their conversion into DSBs during DNA replication. While healthy cells with intact HR mechanisms can withstand PARP inhibition due to their ability to repair DNA through HR, cancer cells with BRCA mutations and impaired HR pathways rely heavily on alternative repair mechanisms such as the low-fidelity repair system non-homologous end-joining, which often results in accumulation of incorrectly repaired DSBs and eventually cell death (5). The simultaneous inhibition of PARP and the HR pathway hampers efficient DSB repair, resulting in synthetic lethality specifically in cancer cells with defective HR pathway. FDA-approved PARPis including olaparib (Lynparza; AstraZeneca), rucaparib (Rubraca; Clovis Oncology), niraparib (Zejula; GSK), and talazoparib (Talzenna; Pfizer) have demonstrated successful clinical outcomes for the treatment of subset of patients with HRD in ovarian, breast, prostate, and pancreatic cancers (6). Several other PARPi are at various stages of preclinical and clinical development as a single agent or in combination with other agents (7).

A key unaddressed challenge with PARPi is the high rate of resistance, approximately 40%–70%, developed by patients over the course of their treatment (8). Cancer cells can develop resistance to PARPi through multiple coping mechanisms such as by the restoration of the HR pathway, reversion mutations leading to reactivation of BRCA1/2, upregulation of alternative DNA repair pathways, modulation of PARP activity, or by upregulation of drug efflux transporters (9). Furthermore, it is well known that responses toward PARPi treatments are heterogeneous (10, 11). Many strategies have been employed to demonstrate the potential to overcome resistance and resensitize tumors to PARPi treatment across various cancer types including combination therapies with new synthetically lethal agents targeting the broader DDR pathways (7). This has led to the emergence of new potential agents targeting ataxia-telangiectasia and Rad3 related (ATR), ataxia telangiectasia mutated (ATM), CHK1/2, WEE1, DNA-PK, POLθ either as monotherapy or in combination with other therapeutics targeting nonoverlapping DDR vulnerabilities. Retrospectively, cisplatin has been identified to belong to the class of drugs where tumors showing maximum platinum agent sensitivity are also the ones characterized by DDR deficiencies (12).

Similarly, acylfulvenes (AF) are categorized as a class of DNA damaging cytotoxins that display advantageous selectivity toward tumors and enhanced effectiveness within cells exhibiting impaired DNA repair capabilities (13). AFs are analogs stemming from naturally occurring sesquiterpenoids called illudins, and their continued development has been aimed at creating cytotoxic agents with refined therapeutic, pharmacokinetic, and safety profiles.

AFs alkylate DNA and form DNA adducts that disrupt DNA and RNA synthesis, induce cell cycle arrest and induce apoptosis (14, 15). The minor groove of DNA was identified as the primary alkylation site where adducts to the 3 position of adenine are formed preferentially (16). It has been reported that DNA lesions induced by illudins and AFs are specifically recognized by transcription-coupled nucleotide excision repair (TC-NER) pathway components, and TC-NER deficiency contributes to the enhanced activity of AFs in numerous solid tumors (17–19). There is an interplay of TC-NER, RNA Pol II as well as HR in the cytotoxic activity of AFs (20). Tumor cell killing by the AF Irofulven depends on the status of BRCA1/2 and Fanconi anemia proteins (FANCD2), which are involved in homology-directed repair of DSBs with Irofulven-induced DSB being considered a replication-associated DNA damage event (21). Overall, while formation of DSBs may be contributing to AF cytotoxicity, the status of HR (or BRCAness), in addition to TC-NER, should be considered as a factor, dictating the expected outcome of treatment with AF analogs.

The fully synthetic small molecule LP-184 (hydroxyurea methylacylfulvene) is a functionalized analog with a foundational AF core structure. LP-184 is specifically activated in tumor cells to an active compound by the oxidoreductase enzyme prostaglandin reductase 1 (PTGR1), that is frequently overexpressed in multiple solid tumor types (22, 23). Apart from the established robust positive association with *PTGR1* expression (24, 25), our current investigation has unveiled novel and distinct correlations between LP-184 sensitivity and components of the HR pathway.

In this study, we investigated antitumor efficacy of LP-184 across a range of HRD solid tumors, including prostate, ovarian, lung, triple-negative breast cancers (TNBC) and LMS both as a standalone treatment as well as in combination with PARPi olaparib by employing established *in vitro*, *ex vivo*, and *in vivo* tumor models.

## Materials and Methods

### Drug Sensitivity and Gene Expression Correlations

**FIGURE 1:**
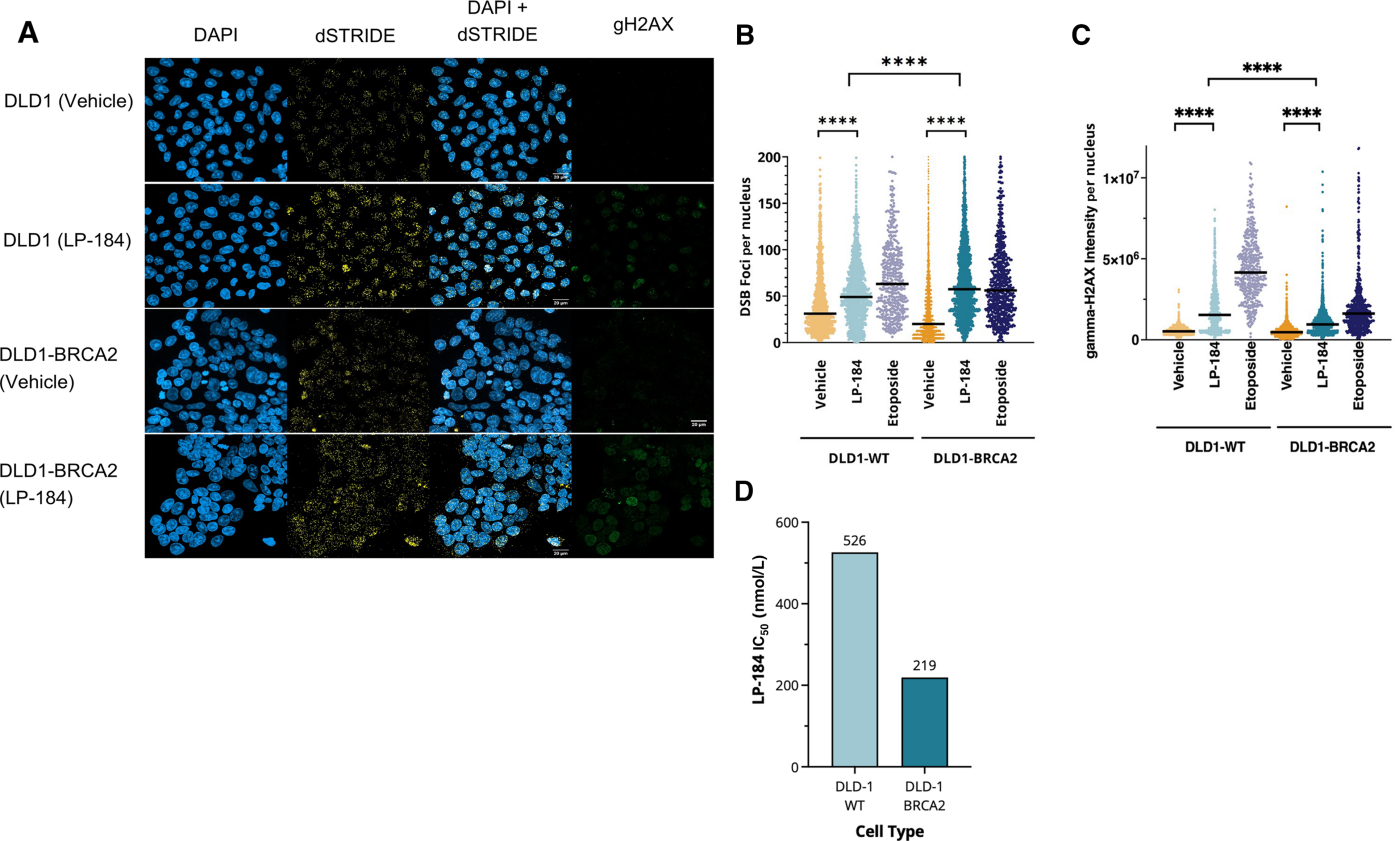
LP-184 induces DNA DSBs and triggers DNA repair. The colon cancer cell line DLD1 and its isogenic *BRCA2* KO cell line DLD1-*BRCA2* were treated with vehicle (0.1% DMSO), 400 nmol/L LP-184, or etoposide for 24 hours. A total of 193,052 nuclei in the vehicle group, 137,489 nuclei in the LP-184 group, and 34,405 nuclei in the etoposide group were surveyed. Double-strand DNA breaks were directly detected using the double-strand DNA breaks with STRIDE (dSTRIDE) technology and DSB foci (yellow) were quantified using an algorithmic approach. Cells were also stained with DAPI (blue), gamma-h2ax (gH2AX, Ser139, red). **A,** Representative images of DSB staining (dSTRIDE) and gH2AX. Each dot represents the number of DSB foci (**B**) or gamma-H2AX foci (**C**) in one nucleus. The median for each group is represented by the short black line. Unpaired Student *t* test was conducted to compare the mean numbers between groups. **D,** LP-184’s IC_50_ were measured in DLD1-WT and DLD1-*BRCA2* using a 72-hour cell viability assay. ****, *P* < 0.001.

Data from CellMiner were used to calculate the correlation between microarray mRNA expression levels and sensitivity of the drugs. In addition to the well-known HR genes, that is, *BRCA1* and *BRCA2*, we selected additional 24 HR genes to find the correlation (26). Pearson correlation method was used to compute the correlation and only the ones with significant *P* value ≤ 0.05 are highlighted in the Fig. 1. Data are available to download from the CellMiner portal (27).

### STRIDE Assay

For the STRIDE assays (28), cells were grown at 37°C with 5% CO_2_ in T25 flasks in DMEM media supplemented with 10% FBS (Sigma-Aldrich) and antibiotics (penicillin and streptomycin). When cells reached about 90% confluency, they were washed with sterile PBS (without Ca^2+^ and Mg^2+^) and incubated for 2 minutes in 1 mL of 0.25% trypsin solution in Ethylenediaminetetraacetic acid (EDTA). Trypsin was inactivated with the culture medium containing FBS and the collected cells were counted in the Bruker chamber. Next, cells were seeded on cover glasses in 12-well plates at a density of 1 × 10^5^ cells/well (passage #19) for DLD-1 wildtype (WT) and 1.5 × 10^5^ cells/well (passage #24) for DLD-1 *BRCA2* knockout (KO) cell line. Cells were then left to rest for 24 hours. Next, the operation medium was replaced with a fresh one containing LP-184 (final concentration 400 nmol/L) or vehicle (0.1% DMSO), and the cells were incubated for 1, 8, and 24 hours. Positive controls were incubated in a medium supplemented with bleomycin solution in DMSO at a final concentration of 12 µg/mL (for sSTRIDE/SSB/pRPA) or etoposide solution in DMSO at a final concentration of 5 µmol/L (for dSTRIDE/DSB/gH2AX). Next, all samples were fixed with ice-cold 70% EtOH and stored at −20°C until the beginning of STRIDE procedures. Finally, dSTRIDE procedure was performed, followed by DAPI (Thermo Fisher Scientific) and phospho-histone H2A.X (Ser139; 20E3) Rabbit mAb [Alexa (R) 488 Conjugate] #20E3 LOT 22 counterstaining (1:100 in 1% BSA for 90 minutes), while sSTRIDE procedure was performed accompanied by DAPI (Thermo Fisher Scientific) and RPA2 (Phospho – S33) CF488A, orb315690 counterstaining (1:100 in 1% BSA for 90 minutes). The coverslips with fixed cells were then mounted with Vectashield (Vector Laboratories) and stored at 4°C until imaging. Fields of view for imaging (at least 10 per sample) were chosen randomly over the surface of the coverslip. The images were then collected as three-dimensional (3D) confocal stacks using Cell Discoverer 7 LSM 900 microscope, Zeiss. γH2AX and pRPA levels were measured as integrated fluorescence from the nuclear region.

### Cell Culture and Cell Viability Assays

Prostate cancer PC3M parental and knockdown cell lines were maintained in modified IMEM (Gibco) supplemented with 10% FBS and 1% penicillin-streptomycin (10,000 U/mL). All cell lines were used within 4–8 passages after being thawed. Cell line authentication was performed and all cell lines were regularly tested for *Mycoplasma* as determined by PCR detection methods. Cells were inoculated into 96-well microtiter plates in 100 µL at a plating density of 5,000 cells/well. After cell inoculation, the microtiter plates were incubated at 37°C with 5% CO_2_ for 24 hours prior to the addition of LP-184. After 24 hours, aliquots of 100 µL of drug dilutions from a 10 mmol/L DMSO stock of LP-184 or olaparib were added to the appropriate microtiter wells already containing 100 µL of medium. Following drug addition, the plates were incubated for an additional 72 hours at 37°C with 5% CO_2_ and the MTT reagent (3-(4,5-dimethylthiazol-2-yl)-2,5-diphenyl-2H-tetrazolium bromide) was used to determine cell viability.

LMS cell lines SK-UT-1B, SK-LMS-1, and SK-UT-1 were maintained in Eagle Minimum Essential Medium with 10% FBS and MES-SA cell line was cultured in McCoy's 5A with 10% FBS in cell culture incubators set at 37°C with 5% CO_2_. Cells were seeded at 800 cells/well in 45 µL of media, incubated for 18 hours, and treated with 0.5% DMSO or LP-184 at various concentrations (6, 10, 30, 60, 100, 300, 600, 1,000, 3,000 nmol/L) for 72 hours in four replicates. After 72 hours treatment, cell viability was measured using Promega's CellTiter-Glo Luminescent Cell Viability Assay.

Colon cancer cell line DLD-1 WT (4,000 cells/well) and DLD-1-*BRCA2* KO (8,000 cells/well) were seeded in triplicates in a flat-bottom black-sided 96-well plate and incubated for 24 hours at 37°C with 5% CO_2_. After the resting period culture medium was replaced with one containing the compound LP-184 in a 3x serial dilution between 2.3 and 5,000 nmol/L in the final volume of 100 µL. After 72 hours of incubation, cell viability was measured using Promega's CellTiter-Fluor assay.

Ovarian cancer cell line OVCAR3 and prostate cancer cell line 22RV1 were maintained in RPMI1640 supplemented with 10% FBS in cell culture incubators set at 37°C with 5% CO_2_. LP-184, olaparib/rucaparib, or LP-184 plus olaparib/rucaparib and DMSO were added the day following cell seeding at various concentrations. On day 5, media was removed and cells received fresh media containing compounds. On day 10, cell viability was measured using Promega's CellTiter-Glo luminescence-based cell viability assay. Drug sensitivity was measured in terms of IC_50_ values generated from dose–response curves plotted in GraphPad Prism version 9. SynergyFinder 3.0 (29) was used to calculate synergy scores.

### Generation of Knockdown Cell Lines

Stable knockdown cell lines were created from prostate cancer cell line PC3M, by transfection with short hairpin RNA (shRNA) targeting various genes involved in DDR. PC3M cell clones deficient in BRCA2, ATM, ERCC2, ERCC3, and ERCC6 at the protein level were generated. PC3M cells with downregulated BRCA2, ATM, ERCC2, ERCC3, and ERCC6 were prepared by transduction with lentivirus expressing gene-specific shRNA from Sigma. Lentivirus was produced in 293T cells and viral particles containing conditioned medium were filtered through 0.45 µm polyvinylidene difluoride membrane and directly used to infect the PC3M cell line in the presence of 8 µg/mL polybrene. A total of 24 hours after infection, cells were selected with puromycin at a final concentration of 3 µg/mL. Surviving cells were pooled together and maintained in a medium containing 1 µg/mL puromycin. Control cells were obtained in a similar manner with lentivirus expressing nontargeting shRNA.

### Generation of Prostate Cancer Organoids

LuCaP 96 and LuCaP 86.2 prostate cancer organoids were derived from patient-derived xenografts (PDX) grown in non-castrate SCID mice. Tumor tissue was harvested and immediately cut into small pieces, 1–2 mm^3^, with a sterile scalpel blade. The tissue was then collected in Advanced DMEM/F12 with 10 mmol/L HEPES and 2 mmol/L Glutamax (Gibco). Tissue fragments were pipetted up and down 50 times with DMEM-F12 media containing 10% FBS and penicillin and streptomycin. Then, it was filtered through a 100 µm sterile cell strainer (Thermo Fisher scientific) and centrifuged at 500 rpm for 5 minutes. This step was repeated three to five times and pelleted small cell clusters were plated on ultra-low attachment culture plates (Nunclon Sphera, Thermo Fisher Scientific) with serum-free culture media (KSFM with EGF, bovine pituitary extract, DHT, and penicillin and streptomycin). The organoids were cultured in a CO_2_ incubator at 37°C.

### Prostate Cancer Xenograft-derived Organoid Viability Assay

Generation one LuCaP organoids were plated on 24-well ultra-low attachment culture plates (Nunclon Sphera, Thermo Fisher Scientific) with KSFM containing 1 nmol/L DHT, and penicillin and streptomycin. Twenty-four hours after plating the media were replaced with fresh media containing LP-184. The total assay duration was 5 days. At the end of this duration, organoid spheres were stained with live and dead cells were labeled with fluorescent reagents Calcein-AM and ethidium homodimer-1 respectively, photographed by fluorescent microscopy, and viable organoids were counted using ImageJ software to yield mean organoid numbers per visualization field. No cells in media were used as the background signal control and 0.3% DMSO alone was used as the vehicle control for data normalization. Mean organoid number/visualization field were plotted and IC_50_ values were generated in GraphPad Prism version 9.

### Patient-derived Tumor Graft Viability Assay

Patient-derived tumor grafts were freshly excised, fragmented, and treated with LP-184 in a 96-well format in triplicate well across nine concentrations ranging from 5.5 nmol/L to 36.45 µmol/L over 5 days. Cell proliferation was measured using the Cell Titer Glo assay. Drug sensitivity was measured in terms of IC_50_ values generated from dose–response curves plotted in GraphPad Prism version 9. Viable cells were metabolically labeled with CellTracker Green, and DNA damage and proliferation were estimated using phosphorylated histone 2AX and 5-ethynyl-2′-deoxyuridine uridine incorporation, respectively. The results were normalized to the nuclei count (Hoechst stain).

#### *In Vivo* Efficacy in TNBC Patient-derived Tumor Xenograft Mouse Study

The TNBC PDXs were sourced from a commercial service provider, Xentech based in France. Human tumor samples were obtained with informed consent from patients treated at cancer centers and established as transplantable xenografts in immunodeficient mice and the study was approved by the local animal review board. The grafted samples are residual material from primary tumors or metastases obtained before or after treatment. These PDX models have been established without prior *in vitro* culture and have been studied for histology, cytogenetics, genetic, and other biological markers, and for their response to standard-of-care (SOC) therapies. The HRD score, RAD51 score, *BRCA1/2* variants, drug response data, and all supporting data and characteristics were obtained from Xentech. An HRD score >50 denotes HR deficiency whereas a score <40 denotes HR proficiency.

Dosing solutions of LP-184 were freshly prepared from powder material by dissolving in ethanol and then adding sterile saline (final concentration being 5% ethanol and 95% saline). Dosing solutions of olaparib were prepared from powder material by dissolving in 10%v/v DMSO in 30%w/v Kleptose (HP-β-CD) in sterile deionized water. Patient-derived TNBC models were grown as xenografts in immune-compromised athymic nude mice. Tumors of the same passage were transplanted subcutaneously into 3 to 24 donor mice, passage (*n* − 1). When these tumors reached a volume of 1,080 to 1,666 mm³, donor mice were sacrificed, tumors were aseptically excised and dissected into fragments measuring approximately 20 mm^3^ and transferred in culture medium before grafting. The tumor fragment was placed in the subcutaneous tissue of the interscapular region in new mouse hosts.

For the study shown in Fig. 4, when tumors reached an average tumor volume of 75 to 221 mm³ animals were randomized into treatment or control groups (*N* = 3 per group) and dosing was initiated on day 0. For each model, animals were administered intravenously every other day × 5on/7off ×2 with (i) 5% ethanol and 95% saline as vehicle for the control group and (ii) 4 mg/kg LP-184 for the treatment group. Dosing occurred on days 0, 2, 4, 6, 8, 16, 18, 20, 22, 24 and animals were monitored for tumor volume and body weight until study termination on posttreatment day 32.

For the study shown in Fig. 6, when tumor volumes reached an average of 60 to 200 mm^3^ animals were randomized into treatment or control groups (*N* = 3 per group) and dosing was initiated on day 1. Animals were treated with vehicle (5% ethanol and 95% saline, intravenously on days 1 and 8 for HBCx-10; intravenously on days 1, 4, 8, and 11 for HBCx-28), LP-184 (intravenously on days 1 and 8 for HBCx-10; intravenously on days 1, 4, 8, and 11 for HBCx-28), olaparib (orally daily on days 1–21 for both HBCx-10 and HBCx-28), or combination of LP-184 and olaparib. Animals were monitored for tumor volume and body weight until study termination on posttreatment day 21 or 22.

Tumors were measured once weekly with in two dimensions using calipers for the duration of the studies (treatment + monitoring), and volume was calculated using the formula: Tumor volume (mm^3^) = *w*^2^ × *l*/2, where *w* = width and *l* = length, in mm, of the tumor. Animal weights were also measured once weekly. Animal behavior was monitored daily.

The mutation status for HBCx-8 is BRCA1 p.Gln81* and for HBCx-10 is BRCA2 p.Gln3036*. These are truncating mutations. Both mutations introduce a stop codon in the sequence resulting in a truncated protein.

The *BRCA1/2*-related LOH is not directly linked to a mutation in these genes, but to the loss of a whole region of the chromosome where the respective gene is located.

The HRD score was determined using the MyChoice CDx scoring from Myriad Genetics. Similarly, the RAD51 score (%) has been used as HRD “like” score. The homologous recombination repair (HRR) functionality was evaluated by an immunofluorescence test. The RAD51 score (%) was quantified as the percentage of geminin-positive cells with 5 or more nuclear foci of RAD51. Geminin staining was used to identify cells in the S–G_2_-phase of the cell cycle, during which the HRR takes place. Cells with RAD51 ≤ 10% were classified as RAD51 low and hence HR deficient (HRD), while cells with RAD51 > 10% as RAD51 high and hence HRR proficient (HRP).

The higher the *BRCA1/2* methylation score for the models, the higher is the promoter methylation status, linked to a HRD.

#### Statistical Analysis

Statistical analysis was performed using GraphPad Prism version 9 unless otherwise specified. Unpaired Student *t* test was used for two group comparisons. Statistical significance was evaluated by *P* values (ns, *P* > 0.05; *, *P* ≤ 0.05; **, *P* ≤ 0.01; ***, *P* ≤ 0.001; ****, *P* ≤ 0.0001).

### Data Availability Statement

The data generated in this study are available upon request from the corresponding author.

### Ethics Reporting

Relevant to the studies using animals and patient samples, we obtained written informed consent from patients, and the studies were conducted in accordance with recognized ethical guidelines, and that the studies were approved by an Institutional Review Board. The authorization to use animals was obtained from The Direction of the Veterinarian Services, Ministry of Agriculture and Food, France.

## Results

### LP-184 Induces DNA DSBs and Triggers HR-dependent DNA Repair

To explore the contribution of DSBs in mediating antitumor cytotoxicity of AF analogs, occurrence of DSBs in human colon cancer DLD1 isogenic cell lines (WT and *BRCA2* KO) in response to LP-184 treatment was evaluated using the STRIDE technology (29). LP-184 treatment led to elevated levels of DSBs and phosphorylation of H2AX (gH2AX). Specifically, LP-184 treatment resulted in a 1.44-fold increase in DSBs in WT DLD1 cells over vehicle (DMSO) control and a 2.46-fold increase in DSBs in *BRCA2* KO DLD1 cells over vehicle (DMSO) control at 24 hours. Following LP-184 treatment, gH2AX was elevated in the nuclei of WT and *BRCA2* KO cell lines. However, it did not strictly correlate with an increase in DSB numbers (Fig. 1; Supplementary Table S1). We also observed increased sensitivity of the *BRCA2* KO DLD1 cells (IC_50_ = 219 nmol/L) compared with the WT DLD1 cells (IC_50_ = 526 nmol/L). Interestingly, only a mild increase in the number of SSBs and phosphorylation of RPA were observed at 24 hours posttreatment with LP-184 when compared with those treated with the vehicle control (Supplementary Fig. S1).

### LP-184 Demonstrated Superior Activity Compared with PARPi Olaparib in Prostate Cancer Cell Line and PDX Organoid Models

**FIGURE 2:**
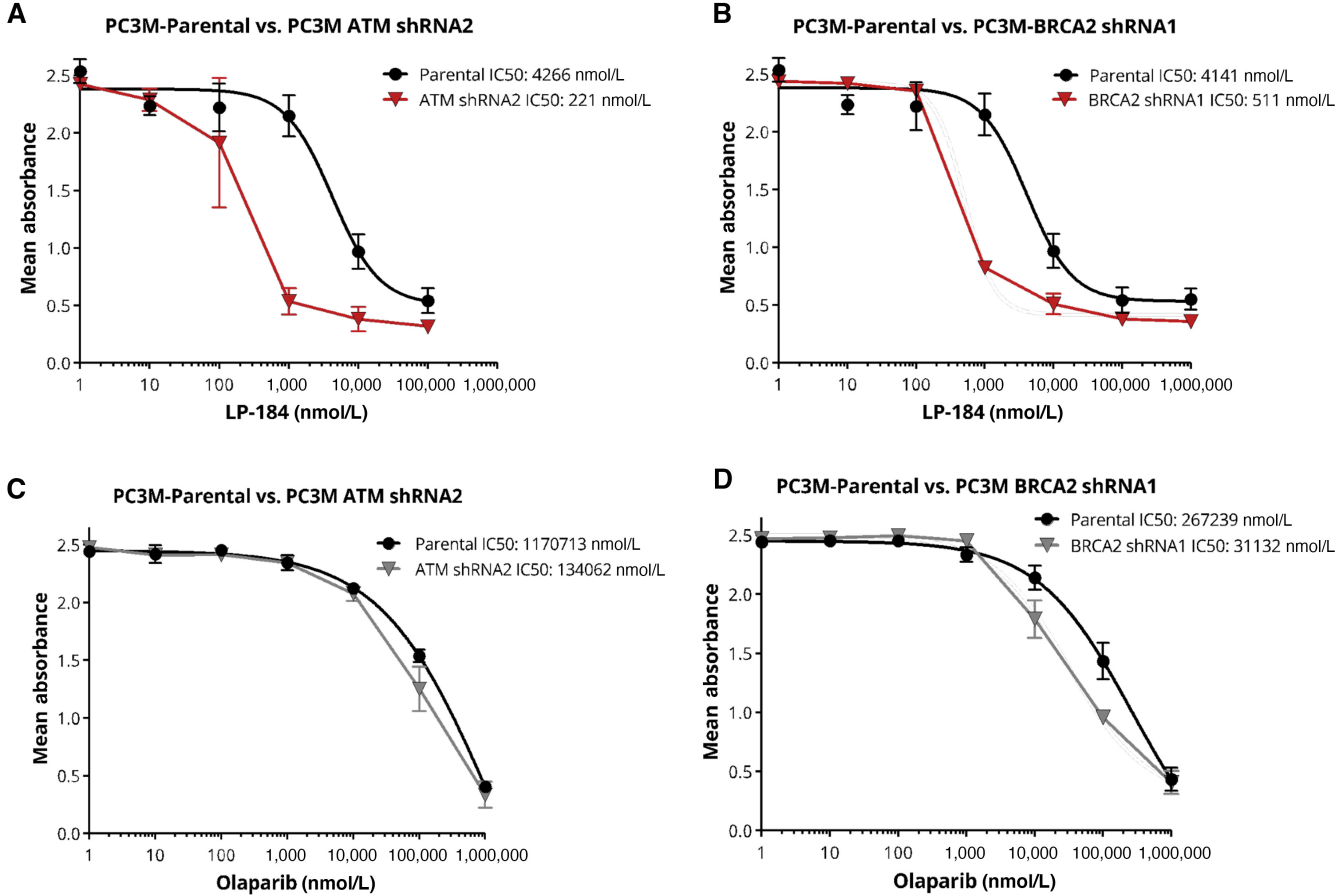
LP-184 response is enhanced in HRD prostate cancer cell lines. Dose–response curves for 3-day treatment of PC3M cells with LP-184 in parental and ATM stable knockdown (**A**), and in parental and BRCA2 stable knockdown (**B**), in comparison with olaparib in parental and ATM stable knockdown (**C**), and in parental and BRCA2 stable knockdown (**D**). Data shown as mean ± SEM.

To investigate the impact of specific HR gene expression on sensitization to LP-184 as a single agent, we selected the highly metastatic prostate cancer cell line PC3M that was resistant to PARP inhibitor (30), to create stable knockdowns of BRCA2 and ATM followed by treating them with LP-184 or PARPi olaparib. As depicted in Fig. 2 (Supplementary Figs. S2 and S3), PC3M cell clones with shRNA mediated knockdown of BRCA2 or ATM showed 8- to12-fold enhanced sensitivity to LP-184 relative to the parental PC3M cell line in a 3-day MTT-based cell viability assay. In the parental PC3M cell line with nontargeted shRNA, LP-184 treatment (IC_50_ ∼4 µmol/L) resulted in 27- to 64-fold increased growth inhibition compared with olaparib (117–267 µmol/L).

**FIGURE 3:**
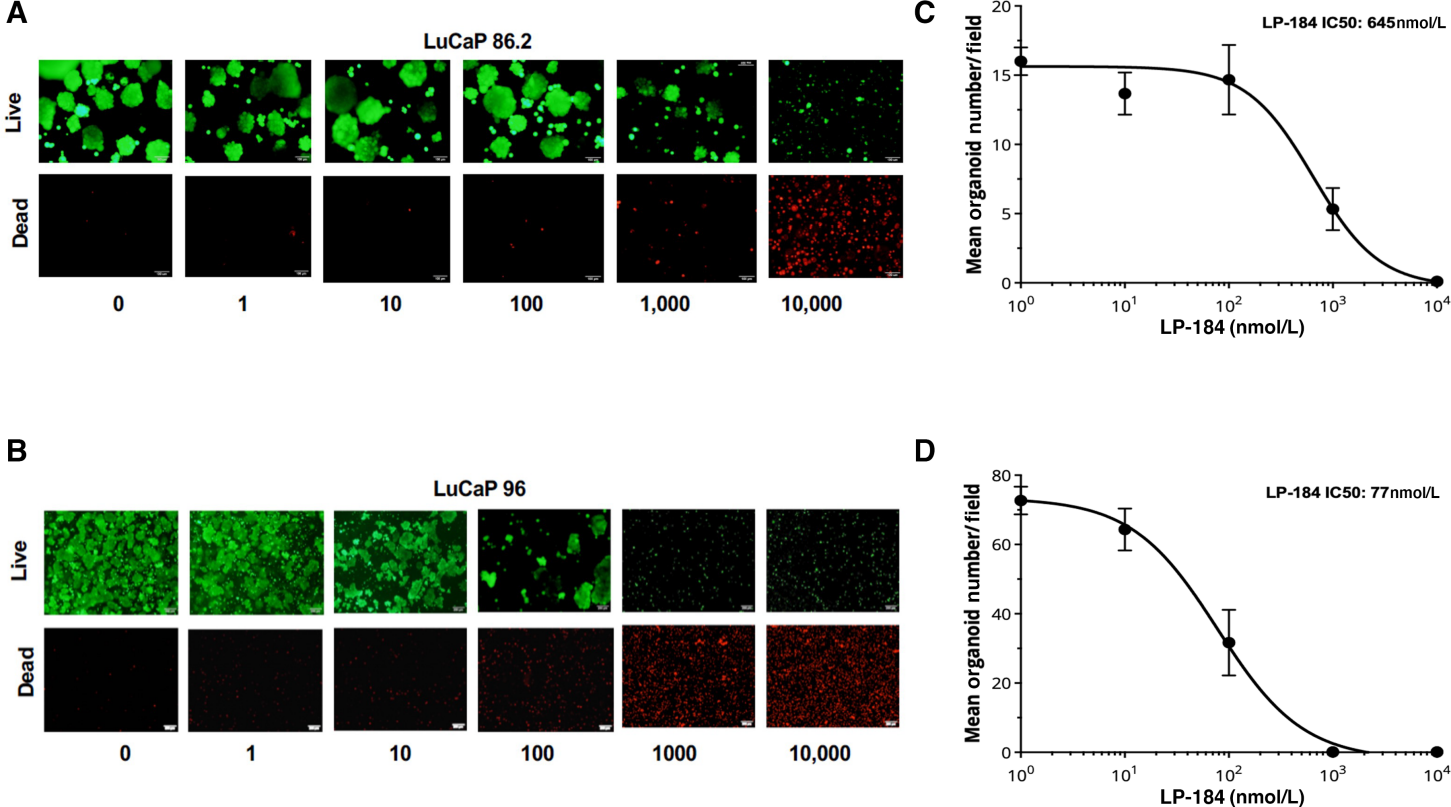
LP-184 is active in LuCaP 96 and LuCaP 86.2 prostate cancer organoids. Fluorescence microscopy images show organoid spheres after 5-day treatment with LP-184 and with Calcein AM staining for live cells and ethidium homodimer-1 staining for dead cells in LuCaP 86.2 (**A**) and LuCap 96 (**B**) organoids. Dose–response curves in LuCaP 86.2 (**C**) and LuCap 96 (**D**) organoids show mean organoid number/field on the *Y* axis and LP-184 test concentrations on the *X* axis.

We compared the sensitivity of LP-184 and PARPi olaparib in prostate cancer organoids derived from previously established PDX models (31). We selected two prostate cancer organoid models, LuCaP 96 and LuCaP 86.2 from a range of castration-resistant prostate cancer PDXs that represent a heterogeneity of clinical specimens. LP-184 demonstrated inhibitory activity in LuCaP 96 (IC_50_ 77 nmol/L) and LuCaP 86.2 (IC_50_ 645 nmol/L) organoids, which was 60- to 120-fold more potent than olaparib in the same models. IC_50_s from a 5-day live/dead cell staining assay performed in these organoid models and associated dose–response curves are displayed in Fig. 3.

We further tested LP-184 activity in patient-derived prostate, lung, and pancreatic tumor graft models characterized by a broad range of mutations in HR pathway genes. *Ex vivo* 3D tumor tissue cultures exhibited a high degree of sensitivity in the range of 30–270 nmol/L IC_50_s in a 5-day viability assay (Table 1) whereas olaparib response across the same models ranged from 720 to 18,000 nmol/L IC_50_s.

**TABLE 1:** LP-184 is effective in patient-derived solid tumor models. LP-184 *ex vivo* potency in a 5-day cell proliferation assay compared with olaparib in a panel of HRD patient-derived tumor graft models representing multiple solid tumor types

Tumor type	Model ID	LP-184 IC_50_ (nmol/L)	Max inhibition (%)	Olaparib IC_50_ (nmol/L)	Max inhibition (%)	Mutated HR genes
Non–small cell lung cancer	CTG-1194	31	91	ND	52	*ATM*
	CTG-2532	54	99	17,000	81	*CHEK1*, *FANCA*, *NBN*, *RAD50*
	CTG-0166	57	97	720	77	*ATM*, *FANCD2*, *NBN*
	CTG-1680	140	99	48,000	88	*PARP2*
	CTG-0192	200	88	2,900	73	*BRCA1*, *RAD54L*
Pancreatic cancer	CTG-1522	45	97	7,900	81	*ATR*, *BRIP1*, *PARP1*
	CTG-1643	57	77	ND	65	*BRCA1*, *BRIP1*
	CTG-0302	110	91	ND	46	*BRCA2*, *ATM*, *BLM*, *FANCA*
	CTG-0314	270	82	1,700	80	*BRCA2*, *CDK12*, *PALB2*
Prostate cancer	CTG-2440	31	95	NR	59	*PMS2*
	CTG-3167	54	97	4,200	48	*BRCA2*, *ATM*, *FANCA*, *FANCI*, *FANCM*
	CTG-3537	54	98	ND	29	*BRCA2*, *CDK12*, *FANCI*, *RAD54L*
	CTG-2429	92	92	18,000	68	*ATM*, *ATR*, *PALB2*
	CTG-3337	230	99	3,700	73	*RAD51C*

Abbreviation: ND: not determined.

### LP-184 Inhibited Proliferation of HRD LMS Cell Lines

Given the strong potency of LP-184 in HRD tumor models derived from prostate, non–small cell lung cancer (NSCLC), and pancreatic cancer, we hypothesized that LP-184 would efficiently target any HRD solid tumor types. LMS is a rare cancer disease with a poor prognosis and limited treatment options. It was reported that over 50% and 98% of patients with LMS carry HR gene mutations and HRD-associated mutational signatures, respectively (4). Therefore, we tested LP-184’s antitumor activities in a panel of four LMS cell lines. According to the report (32), functional assays indicated that SK-UT-1B, SK-LMS-1, and SK-UT-1 LMS cell lines had diminished HR activities. As expected, these three LMS cell lines displayed a remarkably high sensitivity toward LP-184 treatment with IC_50_s ranging between 45 and 173 nmol/L. However, the HR status of the fourth cell line MES-SA was unknown. MES-SA was resistant to LP-184 with an IC_50_ above 3,000 nmol/L, which could possibly be attributed to the low expression of LP-184’s activation enzyme PTGR1 (Table 2).

**TABLE 2:** LP-184 IC_50_ in LMS cell lines. IC_50_ was calculated from 72-hour cell viability assay. PTGR1 RNA expression was retrieved from the DepMap Public 23Q2 Primary Files. HR activity of these cell lines was reported in ref. 34

Cell Line	LP-184 IC_50_ (nmol/L)	PTGR1 RNA expression (log_2_TPM+1)	HR activity
SK-UT-1B	45	NA	Low
SK-LMS-1	135	6.7	Low
SK-UT-1	173	5.8	Low
MES-SA	>3,000	1.02	NA

### LP-184 Exhibited Superior Potency as Compared with Olaparib in TNBC PDX Models that Carry HR Mutations Including PARPi Resistance

**FIGURE 4:**
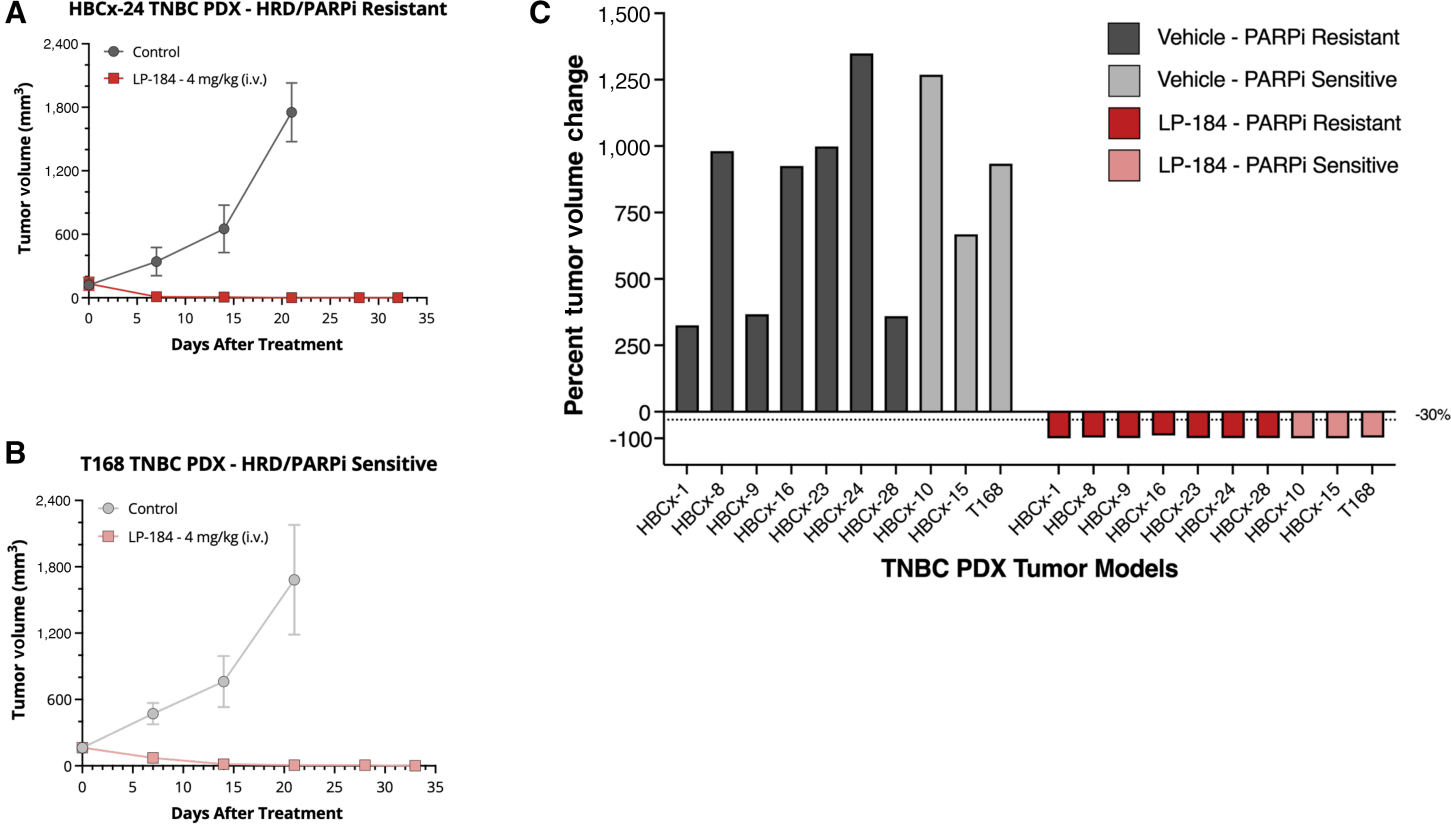
LP-184 is effective in HRD TNBC PDX models regardless of their sensitivity to PARPi. LP-184 treatment (4 mg/kg i.v.) on days 0, 2, 4, 6, 8 and 16, 18, 20, 22, 24 in HRD TNBC PDX models showing HBCx-24 (PARPi resistant) tumor growth curves (mean ± SD) for vehicle versus treatment arms (**A**), T168 (PARPi sensitive) tumor growth curves (mean ± SD) for vehicle versus treatment arms (**B**), and Waterfall plot illustrating tumor regression in all 10 LP-184–treated PDX models (**C**).

Additional *in vivo* evaluation of antitumor activity of LP-184 as a single agent was conducted in a panel of 10 TNBC subcutaneous PDX models established in nude mice. PDXs were derived from primary tumors of 10 patients with treatment-naïve HR deficient (HRD score > 50) TNBC with known *BRCA1/2* LOH. Supplementary Table S2 displays the characteristics of primary TNBC PDXs selected for evaluating *in vivo* antitumor efficacy of LP-184. Seven of 10 models were PARPi resistant whereas three of 10 models were PARPi sensitive. Treatment with LP-184 over days 0–24 in two cycles [4 mg/kg i.v., (every 2 days × 5 then 7 days off)×2] led to complete and durable regression in all 10/10 TNBC HRD-PDX models tested as compared with control (*P* < 0.0001) reaching a day 32 tumor growth inhibition range of 107%–141% whereas seven of 10 models progressed on PARP inhibitor olaparib. Figure 4 (Supplementary Fig. S4) shows representative tumor responses to LP-184 in a PARPi-resistant model and in a PARPi sensitive model, along with a waterfall plot highlighting tumor regression observed in all LP-184–treated models. Furthermore, LP-184 treatment: control tumor volumes (T/C) at the control group end day was 0% in 10/10 models, whereas across the same models for olaparib, T/C was 0% in two of 10 models and ranged from 15% to 90% in 8/10 models. All treatments with LP-184 were well tolerated with only a transient weight loss up to 5% across all models. In summary, LP-184 exhibited superior efficacy as monotherapy in a range of TNBC PDX models that carry HR pathway mutations including PARPi-resistant models.

### LP-184 Demonstrated Strong Synergy with PARP Inhibitors in Ovarian and Prostate Cancer Cell Lines and TNBC PDX Models

**FIGURE 5:**
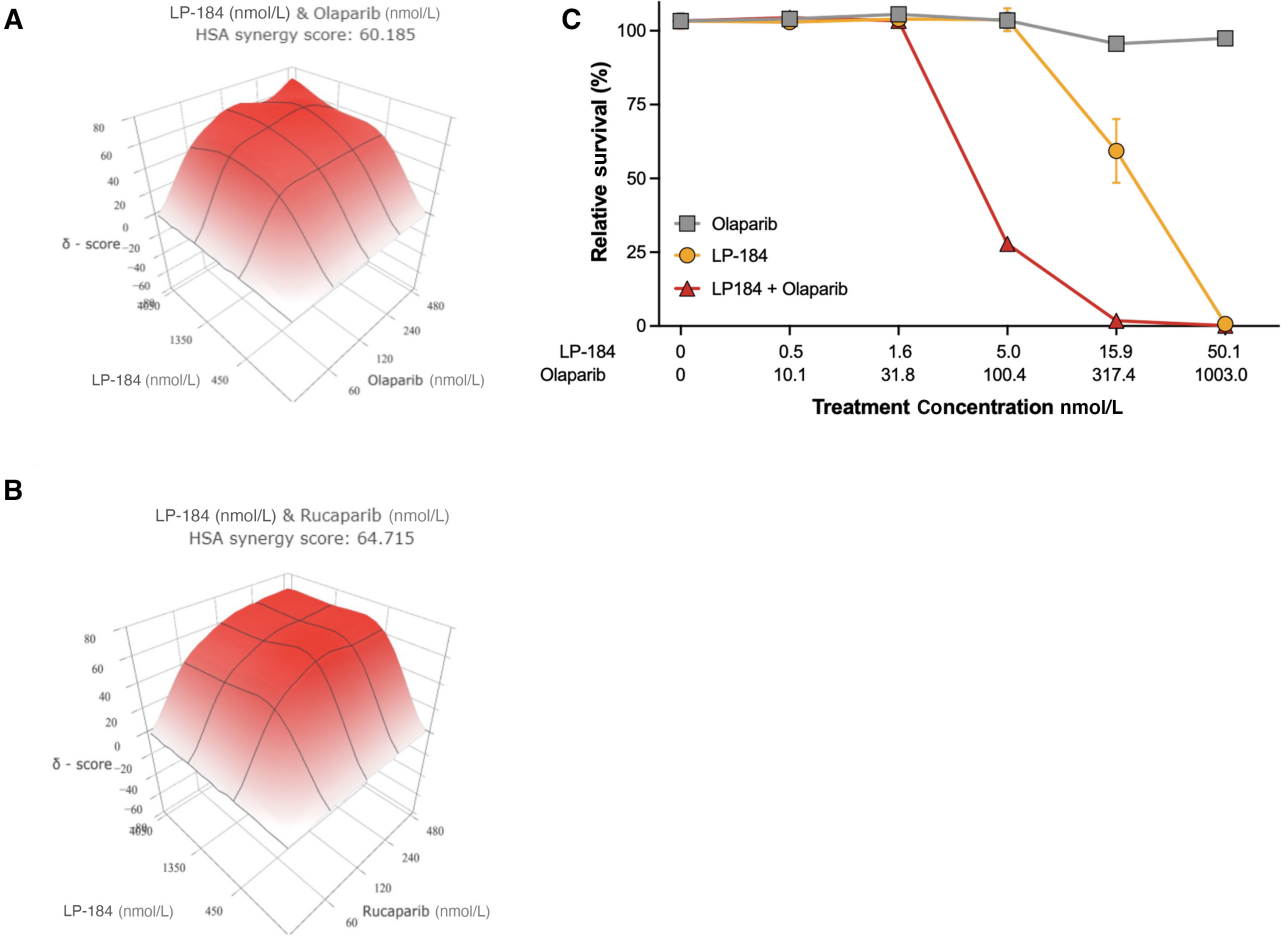
LP-184 and PARPi combination showed strong *in vitro* synergy. **A** and **B,** LP-184 and olaparib/rucaparib synergy in the HRD prostate cancer cell line 22RV1. Cells were treated by 5*4 combinations of LP-184 (0, 60, 120, 240, 480 nmol/L) and olaparib/rucaparib (0, 450, 1,350, 4,050 nmol/L) for 10 days. Cell viability at each combination was used as input for SynergyFinder 3.0 (29) to generate the graphs. **C,** Treatment of LP-184 as a single agent and in combination with olaparib in OVCAR3 cell line. OVCAR3 cells were treated by combinations of LP-184 and olaparib at various concentrations as shown for 10 days. Cell survival was normalized by the viability of untreated cells.

As PARPi sensitizes cells to DNA damaging agents (33), it is possible that PARPi would also potentiate the antitumor activities of LP-184. In the *BRCA2*-mutated 22RV1 prostate cell line, the combination of LP-184 and olaparib resulted in a highest single agent (HSA) score of 60. A similar synergistic effect was observed between LP-184 and another PARPi rucaparib with an HSA score of 65. We then tested whether LP-184 and PARPi combination would be synergistic in other cancer types. The ovarian cancer cell line OVCAR3 had no HR gene mutation but displayed a low level of HR activity as measured by the plasmid rejoining assay (34). As shown in Fig. 5, up to 1,000 nmol/L olaparib alone had nearly no impact on cell viability. However, combining 100 nmol/L olaparib with a nontoxic level of 5 nmol/L LP-184 resulted in 25% cell survival. In addition, 15 nmol/L LP-184 alone killed around 40% of OVCAR3 cells, but adding a nontoxic level of 317 nmol/L olaparib led to nearly 100% cancer cell killing.

**FIGURE 6:**
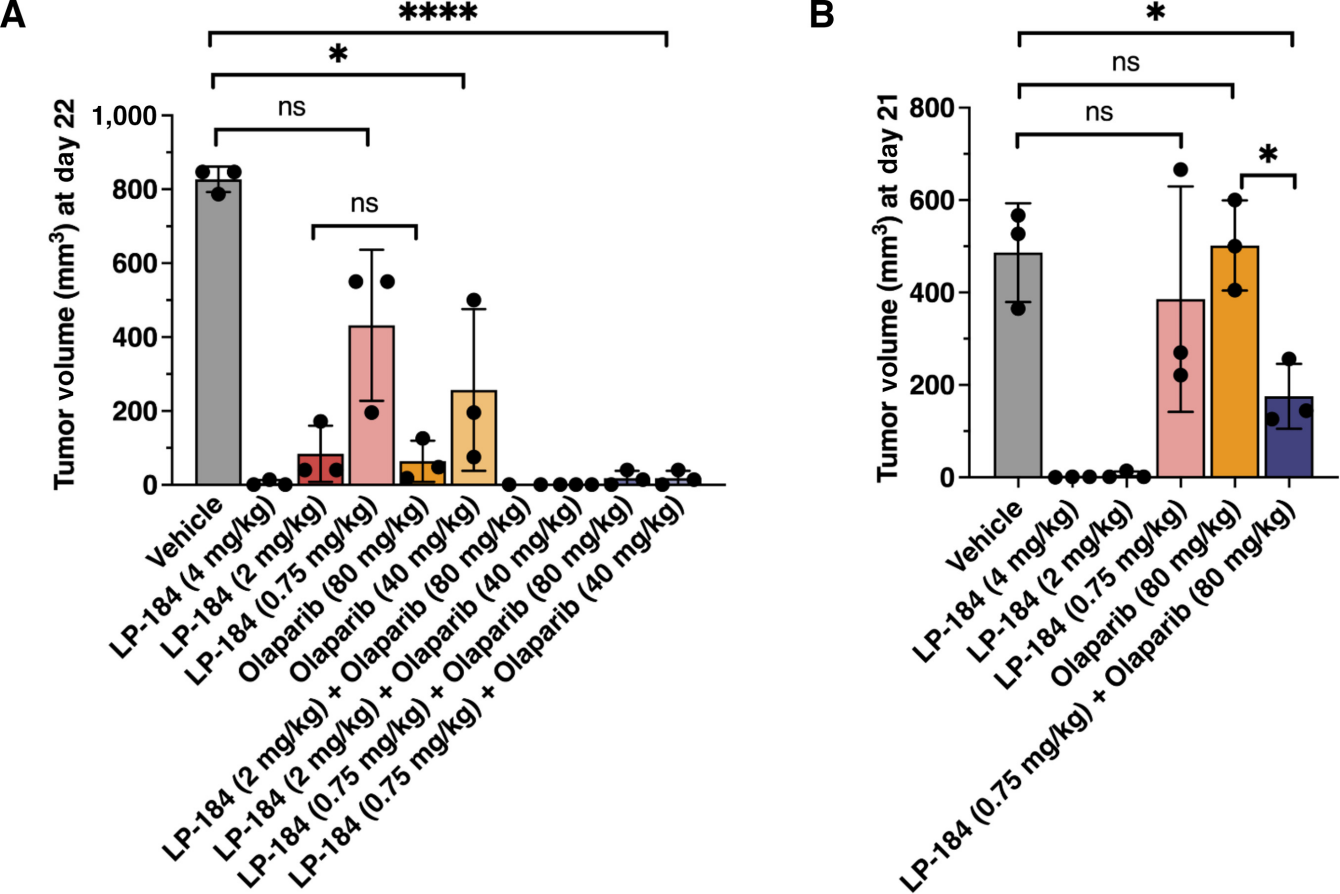
LP-184 and Olaparib combination showed synergy in TNBC PDX models. **A,** The BRCA2-mutated HBCx10 TNBC PDX mouse tumor models were treated with the vehicle (saline), LP-184 (intravenously on days 1 and 8), olaparib (orally daily), or combination of LP-184 and olaparib. Tumor volumes at day 22 were displayed as mean ± SD (*N* = 3 mice/arm). **B,** The BRCA1-mutated HBCx28 TNBC PDX mouse tumor models were treated with the vehicle (saline), LP-184 (intravenously on days 1, 4, 8, and 11), olaparib (orally daily), or combination of LP-184 and olaparib. Tumor volumes at day 21 were displayed as mean ± SD (*N* = 3 mice/arm). Group means were compared using the unpaired *t* test. ns, not significant. *, *P* ≤ 0.05; ****, *P* ≤ 0.0001.

We further interrogated combination effects of LP-184 and olaparib *in vivo*. The PARPi olaparib is currently approved for the treatment of TNBC harboring germline mutations in *BRCA1/2*, but the treatment with olaparib does not yield a response in 40% of patients (35). We therefore tested whether combining LP-184 with olaparib would result in increased tumor inhibition in TNBC PDX models. Briefly, mice implanted with HBCx-10 (*BRCA2*-mutated) or HBCx-28 (*BRCA1*-mutated) TNBC PDX tumors were treated with vehicle (saline), 4, 2, or 0.75 mg/kg body weight of LP-184 (intravenously), 80 or 40 mg/kg body weight of olaparib (orally), or combination of LP-184 and olaparib (Fig. 6). In the HBCx-10 model, at day 22 post treatment initiation, while vehicle-treated mice had an average tumor volume of 827 mm^3^, 4 mg/kg LP-184 treatment led to near complete tumor remission. A total of 2 mg/kg LP-184 treatment resulted in an average tumor volume of 84 mm^3^, comparable with those treated with 80 mg/kg olaparib (*P* = 0.74). In mice treated with lower doses of LP-184 (0.75 mg/kg) and/or olaparib (40 mg/kg), monotherapy resulted in an average tumor volume of 432 mm^3^ (*P* = 0.07 when compared with the vehicle group) and 257 mm^3^ (*P* = 0.04 when compared with the vehicle group), respectively. Interestingly, the combination of 0.75 mg/kg LP-184 and 40 mg/kg olaparib led to an average tumor volume of 18 mm^3^ (*P* = 3.1e-05 when compared with the vehicle gorup), indicating synergism. The trend of synergism was also observed in the *BRCA1*-mutated HBCx-28 model. At day 21 posttreatment initiation, the vehicle-treated mice had an average tumor volume of 486 mm^3^. While 0.75 mg/kg LP-184 monotherapy and 80 mg/kg olaparib monotherapy resulted in an average tumor volume of 385 mm^3^ (*P* = 0.56 when compared with the vehicle group) and 501 mm^3^ (*P* = 0.86 when compared with the vehicle group), respectively, the combination of both led to an average tumor volume of 175 mm^3^ (*P* = 0.02 when compared with the vehicle group). It is also noteworthy that near complete tumor remission was observed in both 4 and 2 mg/kg LP-184–treated HBCx-28 models. The tumor growth curves over the treatment period are shown in Supplementary Fig. S5.

### LP-184 Demonstrated Correlation with Higher Number of HR Pathway Genes as Compared with Cisplatin

In our previous study, we conducted a gene set enrichment analysis on genes associated with LP-184 and found a significant negative enrichment score for DDR pathways, which suggests that genes that are inversely related to LP-184 activity are proficient in mode of action involved in DDR (36). To further investigate the relationship between increased LP-184 activity and DDR deficiency, we examined how the expression levels of established HR genes correlate with LP-184 sensitivity. We explored the transcript expression levels of genes associated with the HR pathway, aiming to identify the most relevant HR deficiency associated with increased LP-184 activity (Supplementary Fig. S6). It is worth noting that the activation of the HR pathway has previously been observed in the context of repairing damage caused by another alkylating agent cisplatin (37). In light of this, we conducted a comparative analysis between LP-184, cisplatin and carboplatin to understand gene expression patterns related to HR pathway components. The *RMI2* gene exhibited a significant negative correlation, and *ATR* and *XRCC2* genes demonstrated marked positive correlations with cisplatin response, *RMI2* exhibited a significant negative correlation also with carboplatin response, whereas eight out of 26 genes exhibited significant negative correlation with LP-184 response with highest negative correlation shown by *DMC1* followed by *BLM* and *XRCC2*. Only the genes that show significant correlation (*P* value ≤ 0.05) are displayed in Supplementary Fig. S6 with their correlation coefficients. Correlations between expression levels of these HR genes with response to other PARP inhibitors Veliparib and Olaparib were variable possibly due to either the short 2-day assay duration that NCI60 screening follows or the correlation of HR genes with PARPi sensitivity being more tightly linked to genomic (DNA mutations) rather than transcriptomic (mRNA expression) profiles.

## Discussion

We present data demonstrating favorable efficacy and synergistic effects of the AF class member, LP-184, in established *in vitro*, *ex vivo*, and *in vivo* tumor models across a range of solid tumors including pancreatic, lung, prostate, TNBC, and LMS carrying deficiency in HR pathway components.

LP-184 possesses a unique mechanism of action by which it is selectively activated in tumors expressing a threshold level of PTGR1 and it is synthetically lethal in DDR tumors (24). LP-184 treatment resulted in an increase in numbers of DSBs as verified through the dSTRIDE assay, which would possibly lead to the observed increased level of gH2AX. Compared with the vehicle treatments in DLD1 WT or *BRCA2* KO cell lines, LP-184 treatment showed apparent accumulation of DSBs and gH2AX signal. These findings support previous reports about Irofulven's ability to trigger HR response (14, 21). DNA damaging agents can induce DSBs directly or through the rapid conversion of SSBs to DSBs during replication. We hypothesize that the latter scenario may be applicable to LP-184. First, AFs are well known to cause TC-NER–dependent single-strand lesions. Second, single alkylating agents barely result in any DSBs in HRP cells (38). Third, we did not observe strong DSB induction by LP-184 at earlier timepoints in either the HRP or HRD cell lines (Supplementary Fig. S1), indicating progression to DSB via cell replication–dependent processes may be a prerequisite. Fourth, we observed PARPi and LP-184 synergy *in vitro* in the prostate cancer cell line 22RV1 and in the ovarian cancer cell line OVCAR3 as well as *in vivo* in TNBC PDX models HBCx-10 and HBCx-28. It is likely that PARPi-mediated retention of PARP1 is triggered by LP-184–induced single-strand lesions, which in turn blocks replication during S-phase and subsequently leads to DSBs after replication fork collapse. In the context of HRD cells, the resulting DSBs are unlikely to be repaired timely and eventually more cell apoptosis would occur. However, we did not observe a higher fold increase of SSBs or pRPA upon LP-184 treatment in our assays, and this could be due to the high level of endogenous SSBs in DLD1 cells making the LP-184–induced SSBs invisible and/or timely repair of LP-184–induced SSBs. There is hence a strong rationale for further investigation of the impact of replication stress on LP-184–induced DSBs.

Regardless of the precise mechanism of LP-184–induced DSBs, we have provided evidence showing that LP-184 outperformed PARPi in HRD tumors. Our data confirmed that shRNA mediated stable knockdown of well-known HR genes BRCA2/ATM in a prostate cancer cell line model led to 8- to 12-fold increased sensitivity toward LP-184, as compared with only 2- to 8-fold increased sensitivity toward olaparib. We employed 14 patient-derived tumor graft models pancreatic, lung, and prostate tumor graft models harboring HR mutations (*BRCA1/2*, *CHK1/2*, *ATM/ATR*, *PALB2*, *PARP1/2*, *RAD51*, *FANCA/B*) to compare LP-184’s potency with PARPi olaparib. LP-184 exhibited significantly superior potency with IC_50_s in the range of 30–300 nmol/L as compared with olaparib's IC_50_ ranging between 1,700 and 6,900 nmol/L in these models. Most of the pathogenic HR mutations were concentrated in *BRCA1/2* and *ATM/ATR* genes across all the represented models. Clinical and translational screening parameters of these models revealed that out of 63 SOC treatment options, only nine showed true tumor regression (i.e., tumor growth inhibition or TGI > 100%) in mouse models. In addition, 19 out of 27 matched patient-treatment pairs with available data did not clinically respond to SOC treatment, with only three out of 27 cases showing confirmed responses without disease progression. This underscores the overall refractoriness of these tumors. The fact that LP-184 demonstrated strong *ex vivo* potency against these refractory tumors supports the general trend of exquisite LP-184 activity in a wide array of HRD cancers.

Similarly, LP-184 outperformed the chemotherapy agent docetaxel in prostate cancer and the PARPi olaparib/niraparib and doxorubicin/cyclophosphamide in TNBC as a single agent in terms of onset as well as duration and robustness of response in PDX models. It is noteworthy that HRD is also being recognized in rare cancers. For example, the rare cancer LMS has a reported HRD percentage ranging between 50% and 98% (4). Our preliminary result showed LP-184’s lower nanomolar potency in HRD LMS cell lines. Further investigation of LP-184’s potency in *in vivo* LMS models would likely provide a promising therapeutic strategy for patients with HRD LMS who otherwise have no effective treatment options available. Our results strongly imply LP-184 can be considered to be a pan-HRD cancer therapeutic agent due to its strong and superior antitumor efficacy as compared with SOC agents in HRD models from various cancer types. There is a possibility to expand HRD targeted therapy using LP-184 in other cancers such as NSCLC and AML (5) as well as subgroups of the rare cancer medulloblastoma carrying germline mutations in PALB2 and BRCA2 (39).

Tolerability in xenograft mouse models tested in this study was characterized in terms of body weight changes on treatment (Supplementary Figs. S7–S10). A total of 4 mg/kg LP-184 treatment as monotherapy resulted in a mean maximum body weight loss of 8%–9% in 2/10 TNBC PDX models and 1%–6% in 8/10 models and considered to lie in an acceptable safety window. Up to 2 mg/kg LP-184 in combination with olaparib resulted in a mean maximum body weight loss of <4% in the two TNBC PDX models tested under the conditions described. All body weight loss trends were fully transient and reversible, and no adverse clinical signs or behaviors were reported for any mouse on study. As comparators, LP-184 IC_50_s in select matched tissue type non-tumor epithelial cell lines from normal pancreas (LP-184 IC_50_ 670 nmol/L) and normal prostate (LP-184 IC_50_ 635 nmol/L) were also tested (Supplementary Table S3). Corresponding dose–response curves have been presented in Supplementary Fig. S11. As described in Table 1, the HRD pancreatic tumor models show mean LP-184 IC_50_ of 120.5 nmol/L and the HRD prostate tumor models show mean LP-184 IC_50_ of 92.2 nmol/L. This demonstrates the potential to achieve 5- to 6-fold differential sensitivity *in vitro* comparing normal cells with inherently HRD cells. Unlike PARP inhibitors, LP-184–driven antitumor cytotoxicity is mediated via two gatekeepers: PTGR1 expression and status of DDR machinery (24). PTGR1 levels are often elevated in solid tumors (23) and in general up to 25% of solid tumors have been analyzed to have some level of DDR deficiency (40, 41). This subset of tumors is predicted to receive the highest benefit from LP-184 therapy. Potential toxicity concerns in normal cells are likely to be mitigated by the retention of intact DNA repair systems in the non-tumor cells. Following initial bioinformatics-driven insights regarding comparative gene correlates of alkylating agent activity, additional experimental evidence on molecular mechanism involving individual HR gene influence on drug activity and treatment effects on a panel of DNA damage and senescence markers would be needed to demonstrate potential advantages of LP-184 over platinum agents.

LP-184 shows superior HRD-targeting single-agent antitumor activity as compared with PARPi. In addition, it might also provide clinical benefit to patient subsets with HRD solid tumors refractory to or ineligible for PARPi treatment. Consistent with reported synergy between PARPi and alkylating agents (42), synergy was observed *in vitro* in multiple cancer types for LP-184 plus PARPi combinations and also *in vivo* in TNBC. The combination of LP-184 and PARPi may offer an attractive therapeutic strategy with likelihood of providing multiple clinical benefits including delaying occurrence of resistance due to targeting non-redundant pathways, and can collaborate with PARPi thus extending the opportunity for utilization of both classes of agents.

Our results illustrate the therapeutic value of LP-184 and support its clinical evaluation in targeting HRD tumors including those that are resistant to PARPi as a single agent in second- or later-line treatment in relapsed/refractory tumors, and in frontline treatment as a combination agent in newly diagnosed/naïve tumors.

## Supplementary Material

Supplementary Figure S1 Figure S1 shows levels of DNA strand breaks in colon cancer cells over 24 hours

Supplementary Table S1 Table S1 shows fold changes in DNA damage markers following LP-184 treatment in vitro

Supplementary Figure S2 Figure S2 shows viability of parental or ATM depleted PC3M cells in response to LP-184 or Olaparib

Supplementary Table S2 Table S2 shows characteristics of the TNBC PDX tumors in which LP-184 efficacy was tested in vivo

Supplementary Figure S3 Figure S3 shows viability of parental or BRCA2 depleted PC3M cells in response to LP-184 or Olaparib

Supplementary Table S3 Table S3 shows LP-184 IC50s in normal cells

Supplementary Figure S4 Figure S4 shows the in vivo tumor response in 8 TNBC PDX models to LP-184 single agent

Supplementary Figure S5 Figure S5 shows the in vivo tumor response in a TNBC PDX model to LP-184 in combination with Olaparib

Supplementary Figure S6. Figure S6 shows a heatmap with correlations between LP-184 activity in NCI0 cancer cell lines and transcript expression levels of Homoloous Recombination genes

Supplementary Figure S7 Figure S7 shows mouse body weight changes in selected TNBC PDX models following LP-184 treatment

Supplementary Figure S8 Figure S8 shows mouse body weight changes in selected TNBC PDX models following LP-184 treatment

Supplementary Figure S9 Figure S9 shows mouse body weight changes in a TNBC PDX model following treatment with LP-184, Olaparib or the combination

Supplementary Figure S10 Figure S10 shows mouse body weight changes in a TNBC PDX model following treatment with LP-184, Olaparib or the combination

Supplementary Figure S11 Figures S11 shows LP-184 activity in normal cells
