# Supplementary material for: LP-184, a Novel Acylfulvene Molecule, Exhibits Anticancer Activity against Diverse Solid Tumors with Homologous Recombination Deficiency
Source: Cancer Res Commun. 2024 May 6;4(5):1199–210. doi: 10.1158/2767-9764.CRC-23-0554 (PMC11072798; doi:10.1158/2767-9764.CRC-23-0554)
Supplement: Supplementary Figure S1 — Figure S1 shows levels of DNA strand breaks in colon cancer cells over 24 hours [file crc-23-0554-s01.docx]

**Supplementary Figure S1**.

(A)

**
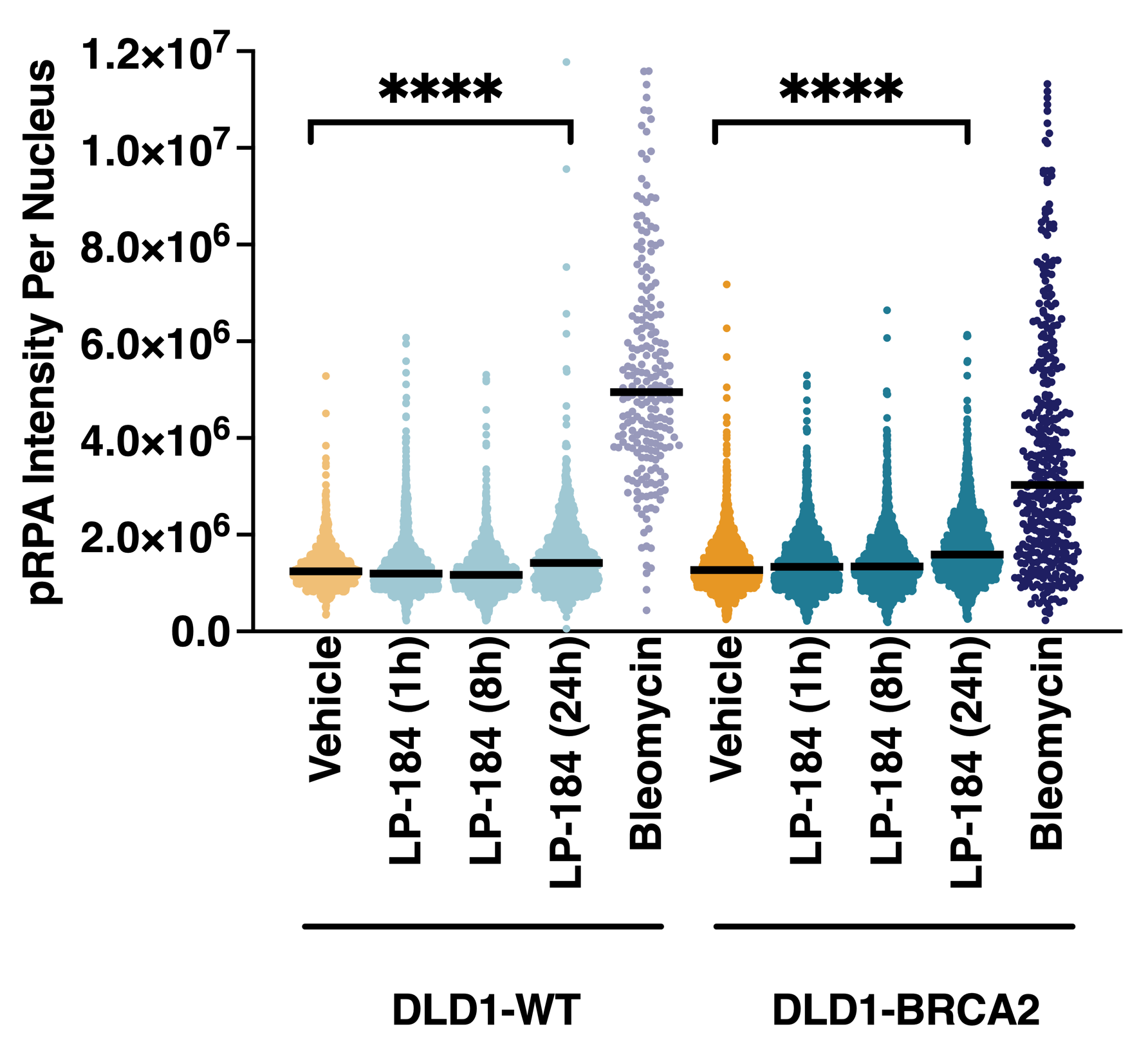

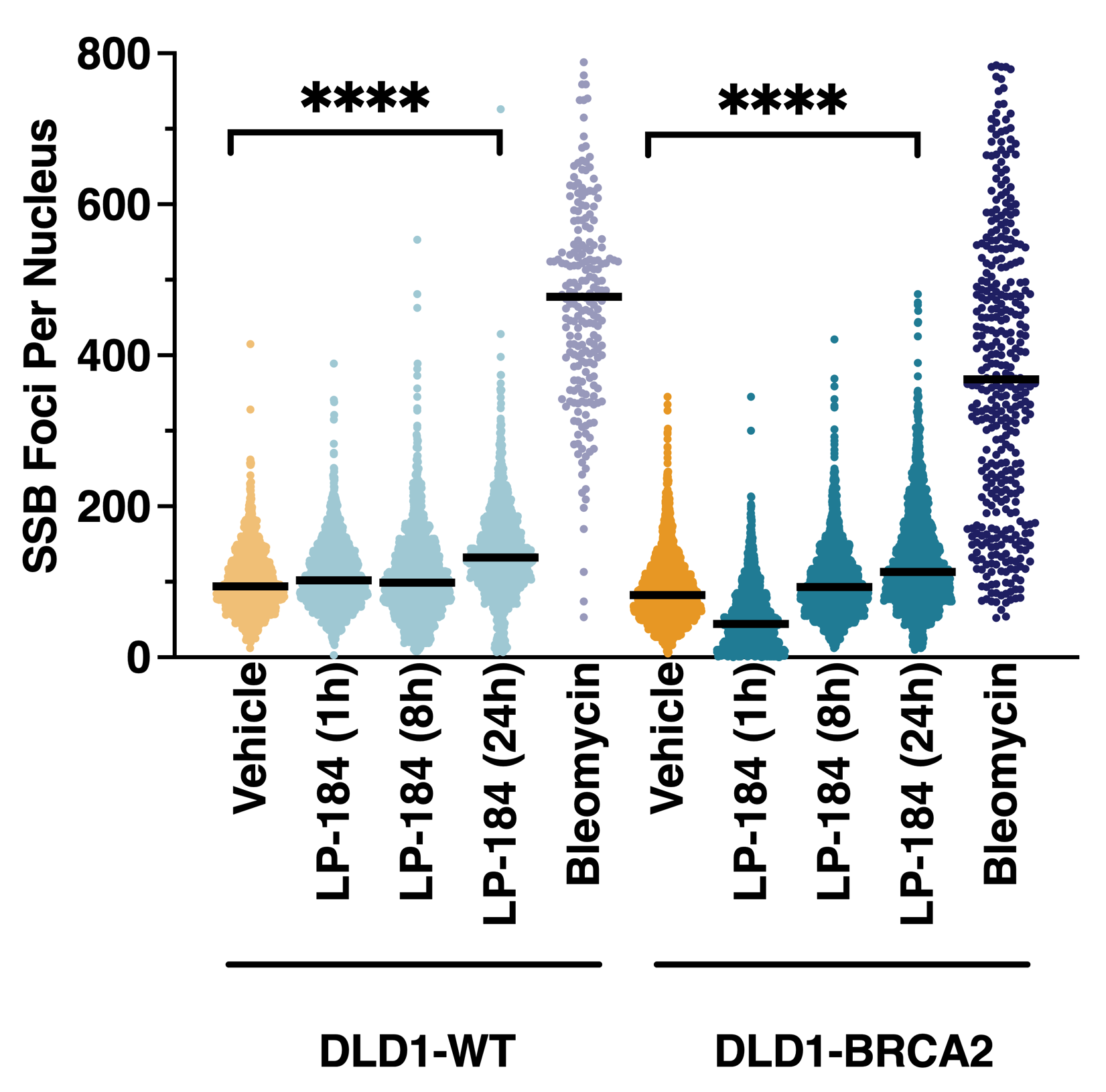
**

(B)

(C)


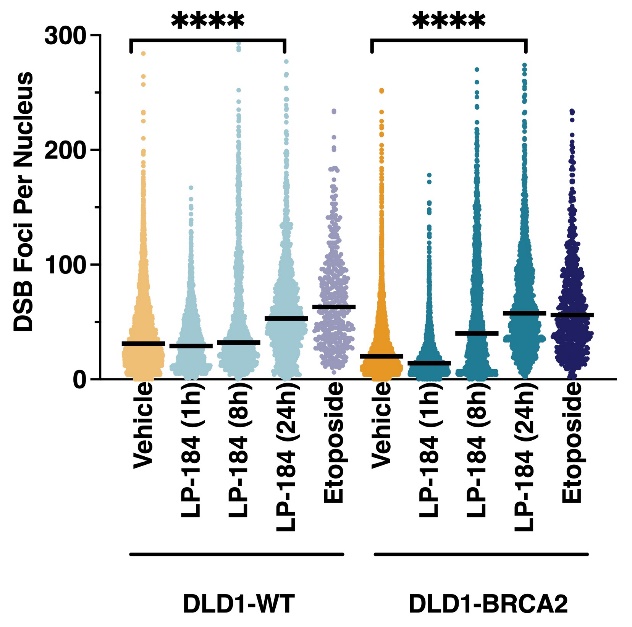


**Figure S1. Temporal effects of LP-184 treatment on DNA strand breaks and associated marker pRPA in DLD1 cells.** Changes in **(A)** SSBs, **(B)** pRPA, and **(C)** DSBs upon treatment with LP-184 at different time points in DLD1 wild type and its isogenic BRCA2 knockout cell lines. The median for each group is represented by the short black line. Unpaired student's t-test was conducted to compare the mean numbers between group. ****, p<0.001.
