# Supplementary material for: LP-184, a Novel Acylfulvene Molecule, Exhibits Anticancer Activity against Diverse Solid Tumors with Homologous Recombination Deficiency
Source: Cancer Res Commun. 2024 May 6;4(5):1199–210. doi: 10.1158/2767-9764.CRC-23-0554 (PMC11072798; doi:10.1158/2767-9764.CRC-23-0554)
Supplement: Supplementary Table S1 — Table S1 shows fold changes in DNA damage markers following LP-184 treatment in vitro [file crc-23-0554-s02.docx]

**Supplementary Table S1.** **Relative changes in DNA damage and DNA repair markers following LP-184 treatment *in vitro*.** Average fold changes of STRIDE assay signals measuring DSBs and SSBs along with related markers gH2AX and pRPA in vehicle or LP-184 treated DLD1 WT or BRCA2 KO colon cancer cells. 5 µM Etoposide was used as the positive control compound.

| **Assay/ condition** | **24h LP-184:vehicle ctrl** | **24h Positive ctrl compound:vehicle ctrl** |
| --- | --- | --- |
| **DSB WT** | 1.44 | 1.85 |
| **DSB BRCA2 -/-** | 2.46 | 2.37 |
| **gH2AX WT** | 3.91 | 9.45 |
| **gH2AX BRCA2 -/-** | 1.29 | 2.90 |
| **SSB WT** | 1.37 | 4.77 |
| **SSB BRCA2 -/-** | 1.41 | 4.33 |
| **pRPA WT** | 1.17 | 4.10 |
| **pRPA BRCA2 -/-** | 1.25 | 2.72 |
