# Supplementary material for: LP-184, a Novel Acylfulvene Molecule, Exhibits Anticancer Activity against Diverse Solid Tumors with Homologous Recombination Deficiency
Source: Cancer Res Commun. 2024 May 6;4(5):1199–210. doi: 10.1158/2767-9764.CRC-23-0554 (PMC11072798; doi:10.1158/2767-9764.CRC-23-0554)
Supplement: Supplementary Figure S2 — Figure S2 shows viability of parental or ATM depleted PC3M cells in response to LP-184 or Olaparib [file crc-23-0554-s03.docx]

**Supplementary Figure S2**.

**
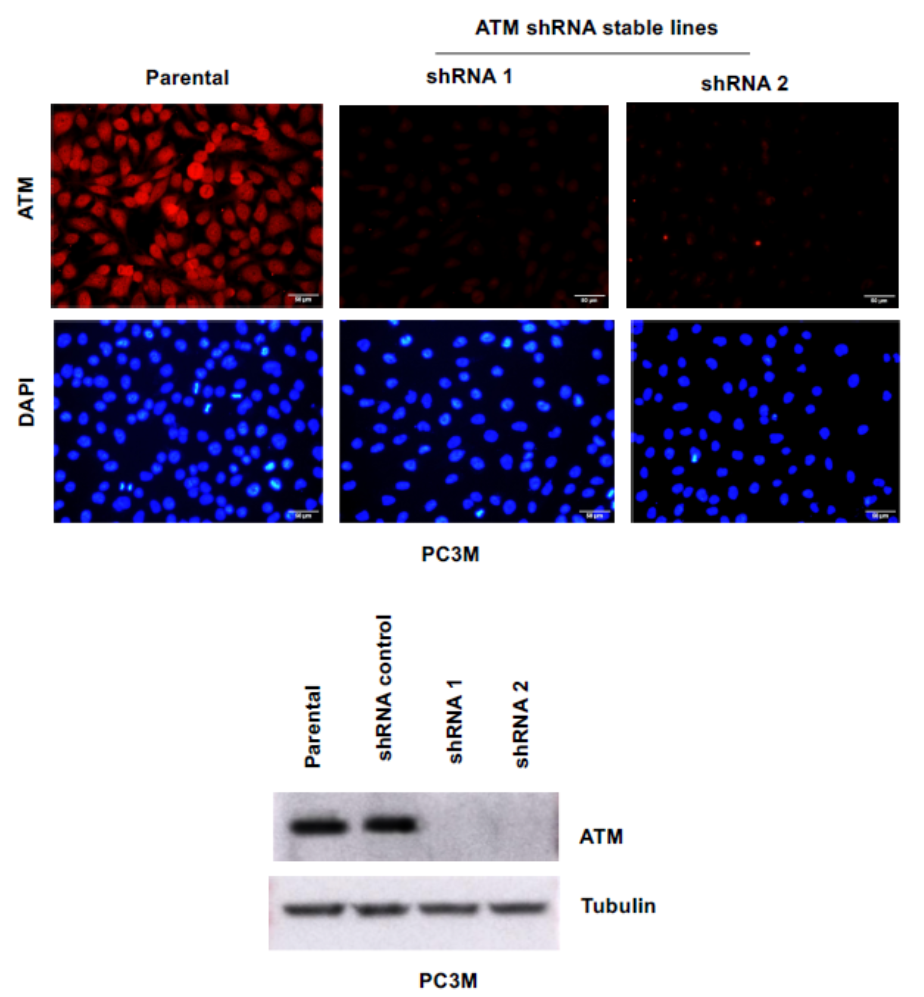

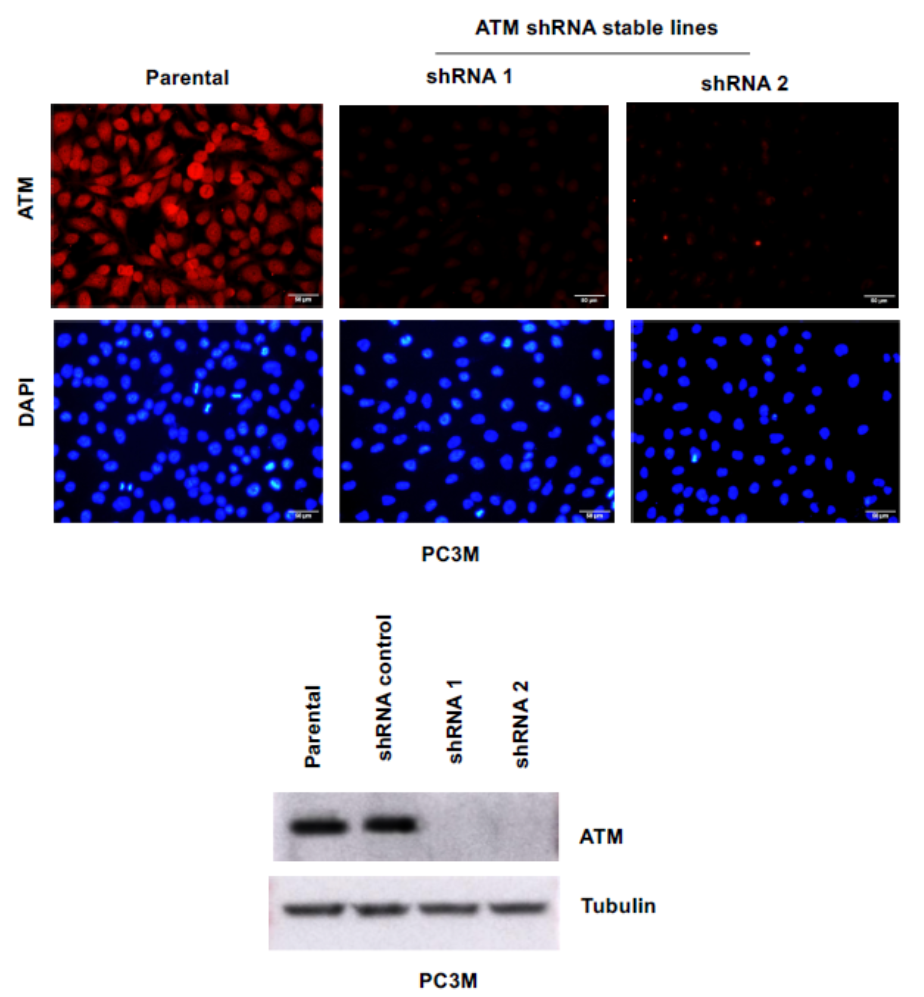

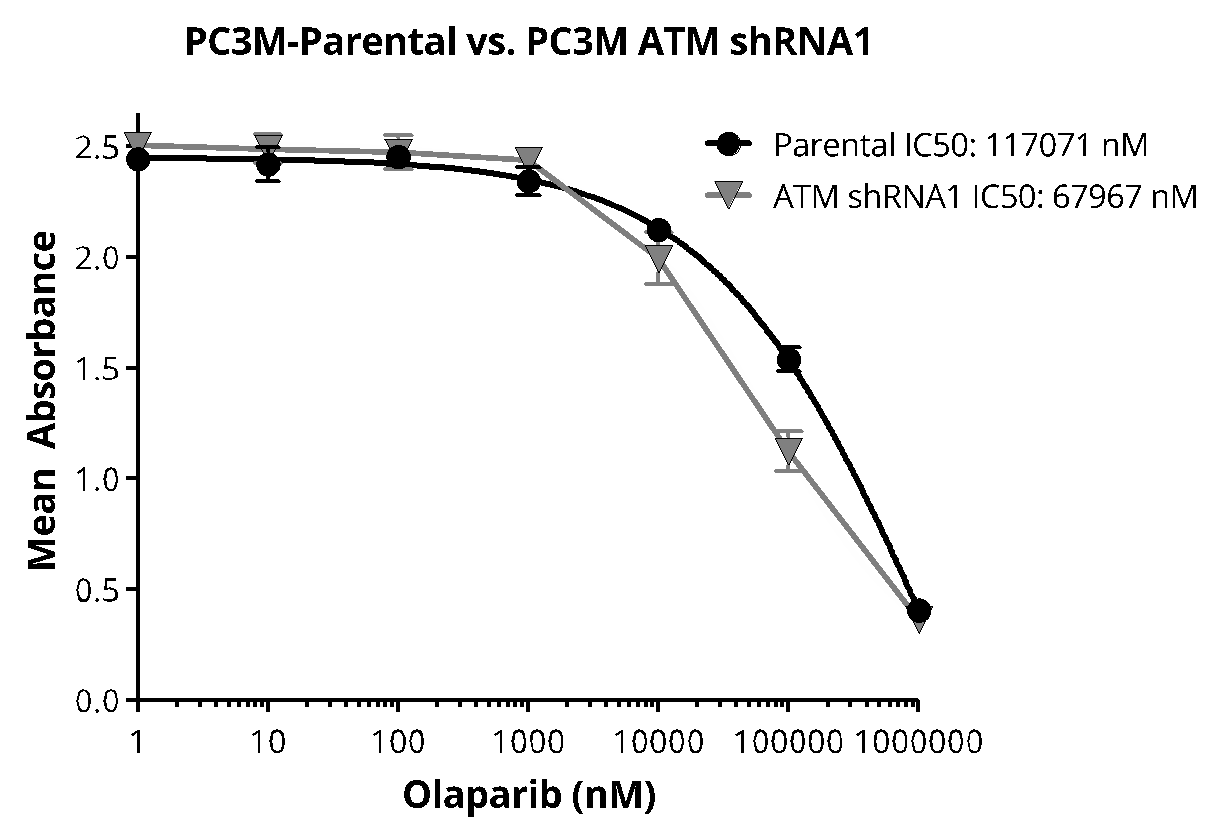

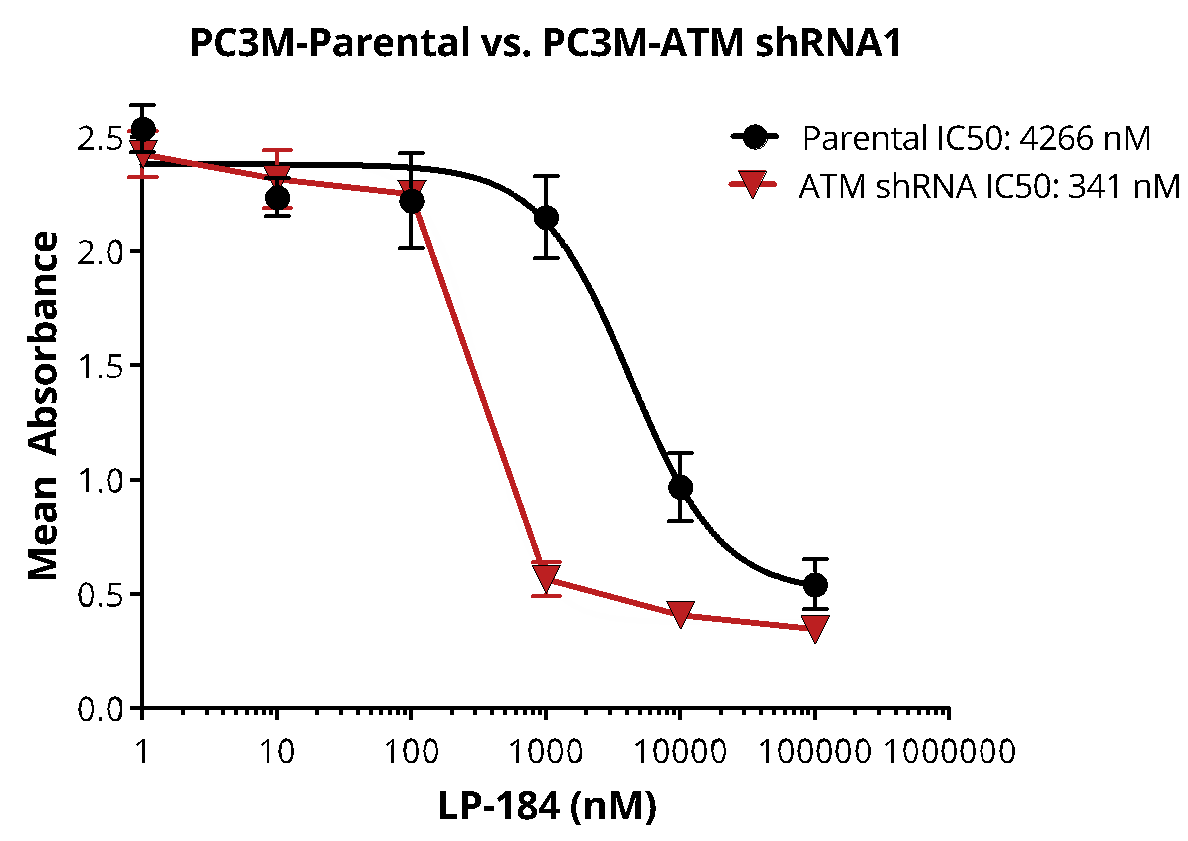
**

(B)

(A)

(C)

(D)

**Figure S2**. **Viability of parental or ATM depleted PC3M cells in response to LP-184 or Olaparib.** PC3M parental and ATM shRNA knockdown stable cell clones **(A)** treated with LP-184 for 3 days, **(B)** treated with Olaparib for 3 days, **(C)** treated with antiATM antibody and nuclear stain DAPI visualized by fluorescence microscopy and **(D)** immunoblotted for ATM and Tubulin (as loading control)
