# Supplementary material for: LP-184, a Novel Acylfulvene Molecule, Exhibits Anticancer Activity against Diverse Solid Tumors with Homologous Recombination Deficiency
Source: Cancer Res Commun. 2024 May 6;4(5):1199–210. doi: 10.1158/2767-9764.CRC-23-0554 (PMC11072798; doi:10.1158/2767-9764.CRC-23-0554)
Supplement: Supplementary Table S2 — Table S2 shows characteristics of the TNBC PDX tumors in which LP-184 efficacy was tested in vivo [file crc-23-0554-s04.docx]

**Supplementary Table S2.** **Characteristics of primary TNBC PDX tumors selected for evaluating LP-184 anti-tumor efficacy *in vivo.*** This table displays disease, treatment and response information of patients from whom xenografts were derived, along with their genomic background.

|  | **TNBC PDX model** | **Prior treatment** | **Lehman classification** | **HRD score (>50: HRD, < 40: HRP)** | **RAD51 score** | **BRCA1/BRCA2 variants** | **BRCA1/BRCA2 LOH** | **BRCA1 promoter methylation** | **PTEN variants (allele frequency)** | **Other relevant variants** | **Response to Adriamycin/ Cyclophosphamide** | **Response to PARPi (Olaparib/ Niraparib)** | **Day 32 tumor growth inhibition (TGI%) with 4 mg/kg LP-184 treatment (2 cycles)** |
| --- | --- | --- | --- | --- | --- | --- | --- | --- | --- | --- | --- | --- | --- |
| HR Deficient + PARPi resistant | HBCx-1 | None | BL1 | 50 | 25.0 | - |  | 9.4 |  |  | S | P | 132.2% |
|  | HBCx-8 | RT | M | 65 | 41.7 | BRCA1 p.Gln81* | BRCA1 | 0.3 |  |  | S | P | 109.9% |
|  | HBCx-9 | None | BL1 | 55 | 33.0 | - | BRCA1 | 13.8 |  |  | P | P | 127.6% |
|  | HBCx-16 | None | M | 57 | 42.0 | - | BRCA1 | 12.1 | ex3-5 del (1) |  | P | P | 119.6% |
|  | HBCx-23 | None | M | 69 | 35.5 | - | BRCA1 | 61.7 |  |  | S | P | 109.9% |
|  | HBCx-24 | None | BL1 | 55 | 38.7 | - |  | 4.3 |  |  | P | P | 107.3% |
|  | HBCx-28 | None | BL2 | 63 | 36.0 | BRCA1 c.212+3A>G [IVS5+3A>G] | BRCA1 | 0.9 | gene loss | SHLD2 loss | S | P | 141.4% |
| HR Deficient + PARPi sensitive | HBCx-10 | None | M | 57 | 1.7 | BRCA2 p.Gln3036* | BRCA2 | 0.5 | ex3 del (1) |  | CR | CR | 107.9% |
|  | HBCx-15 | None | MSL | 68 | 2.7 | - | BRCA1 | 33.9 |  | STK11 loss | CR | CR | 127.1% |
|  | T168 | None | BL1 | 54 | 1.7 | BRCA1 p.Ser1524Leufs*24 | BRCA1 | 0.0004 |  |  | CR | CR | 110.4% |

RT: radiation therapy, BL1: basal-like 1, M: mesenchymal, BL2: basal-like 2, MSL: mesenchymal stem-like, S: stable disease, P: progressive disease, CR: complete response
