# Supplementary material for: LP-184, a Novel Acylfulvene Molecule, Exhibits Anticancer Activity against Diverse Solid Tumors with Homologous Recombination Deficiency
Source: Cancer Res Commun. 2024 May 6;4(5):1199–210. doi: 10.1158/2767-9764.CRC-23-0554 (PMC11072798; doi:10.1158/2767-9764.CRC-23-0554)
Supplement: Supplementary Figure S3 — Figure S3 shows viability of parental or BRCA2 depleted PC3M cells in response to LP-184 or Olaparib [file crc-23-0554-s05.docx]

**Supplementary Figure S3**.

**
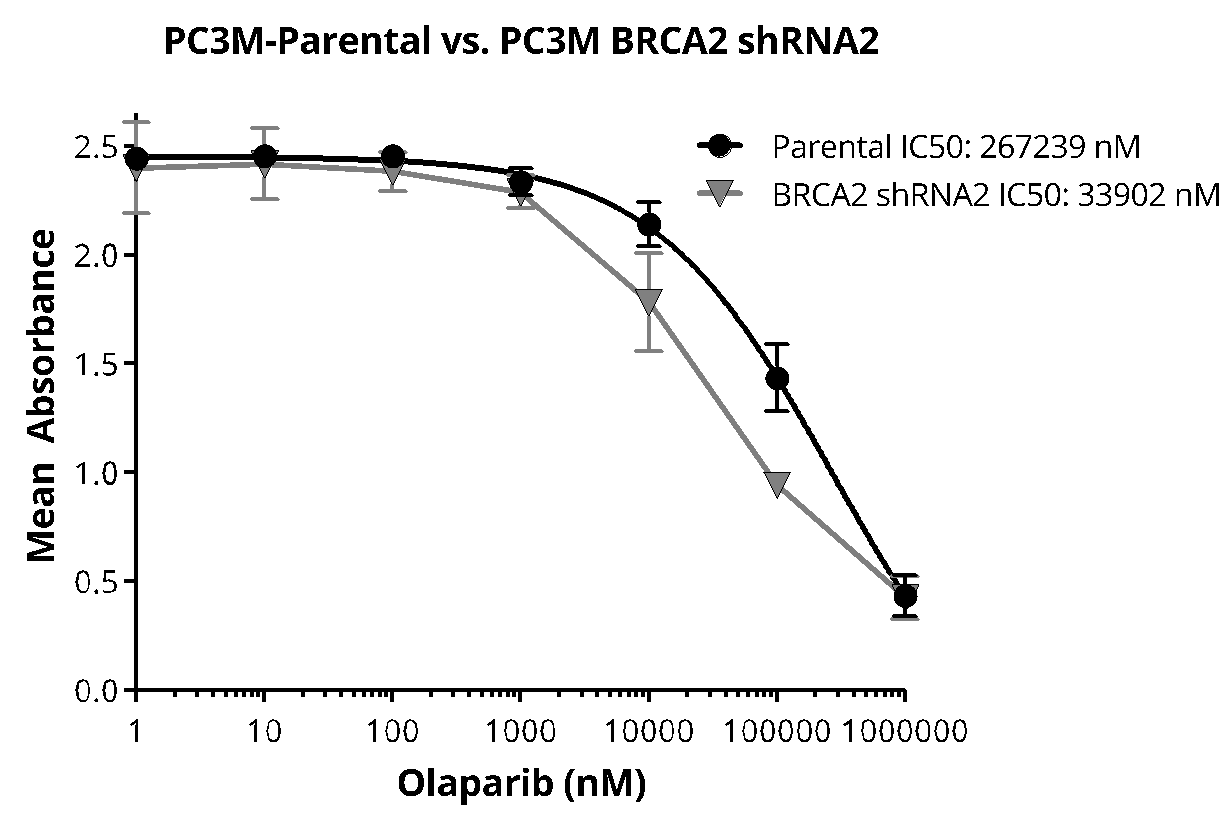

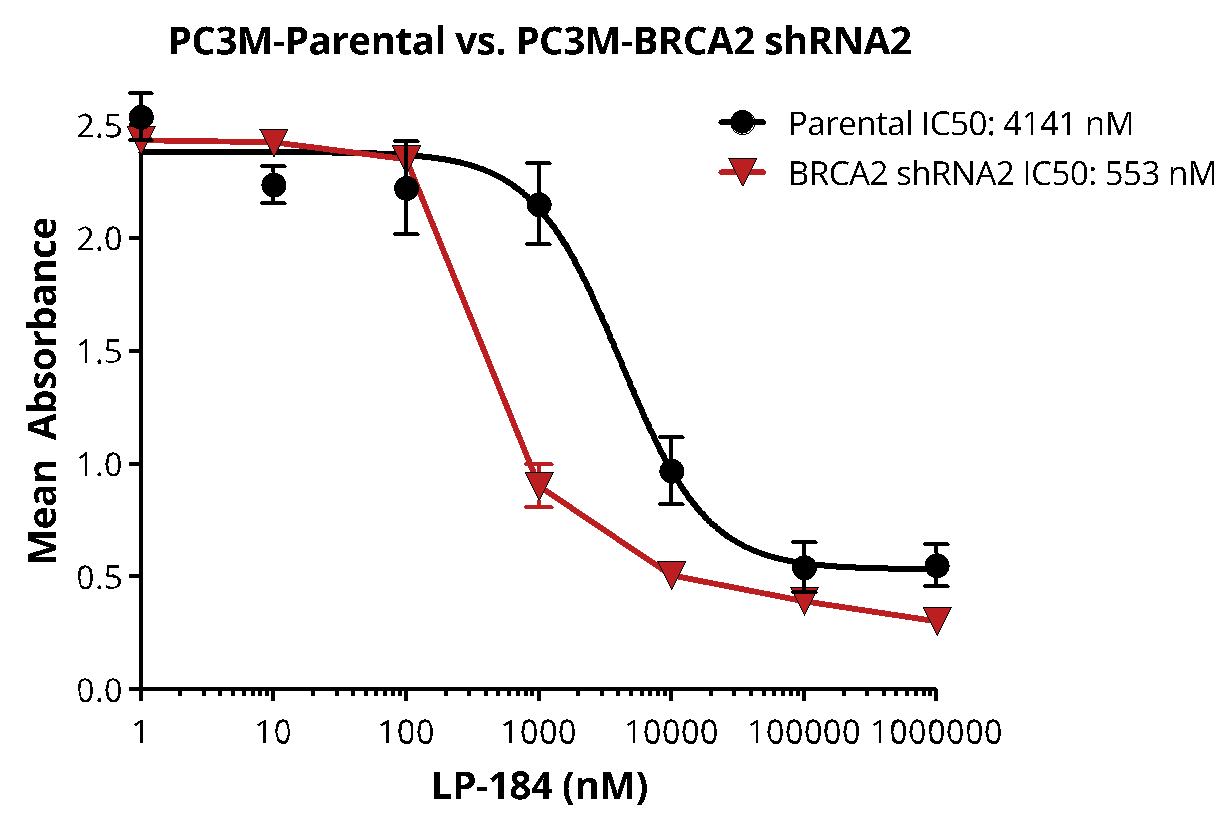

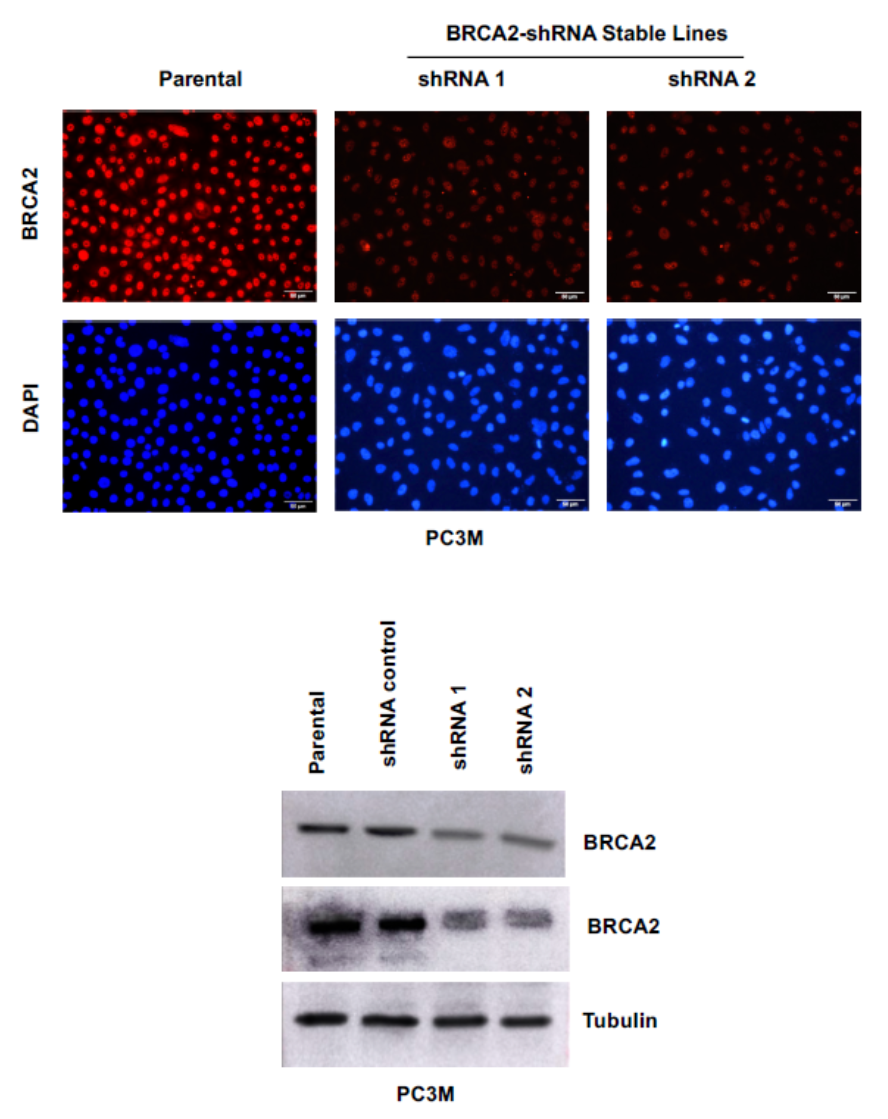
**

(B)

(A)


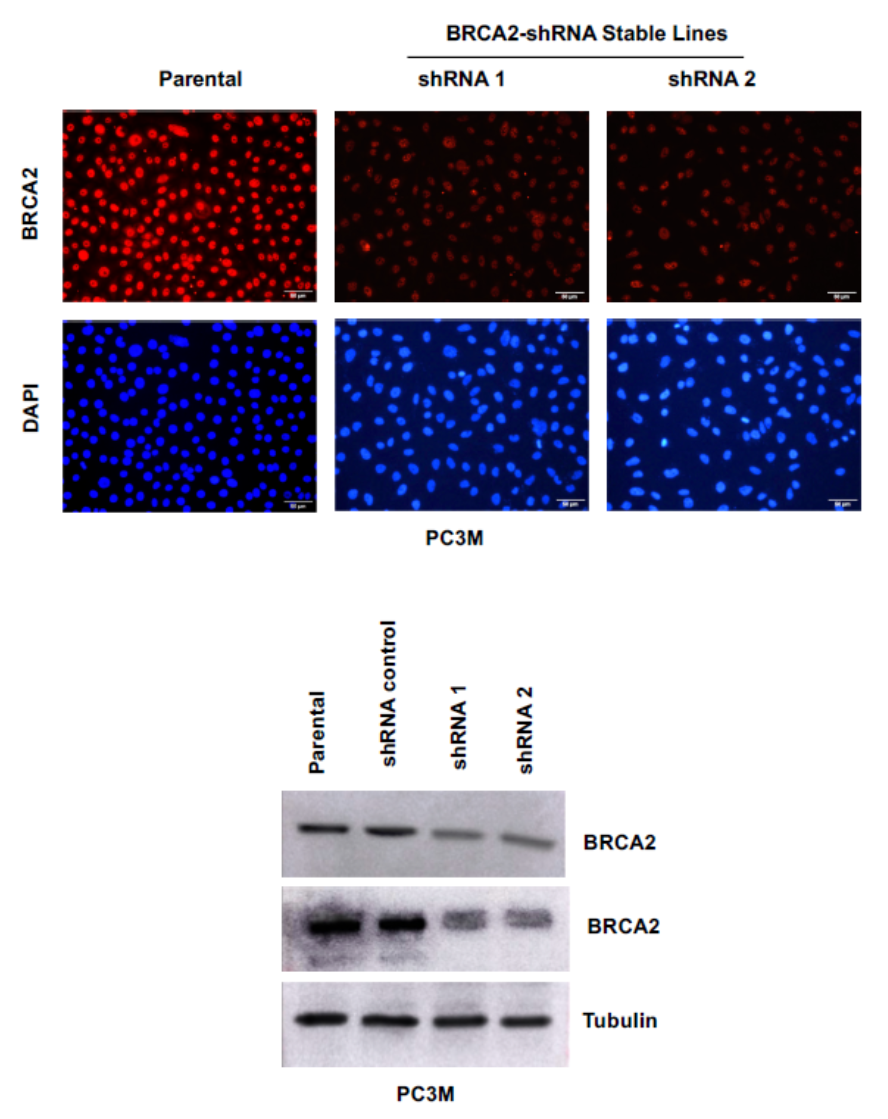

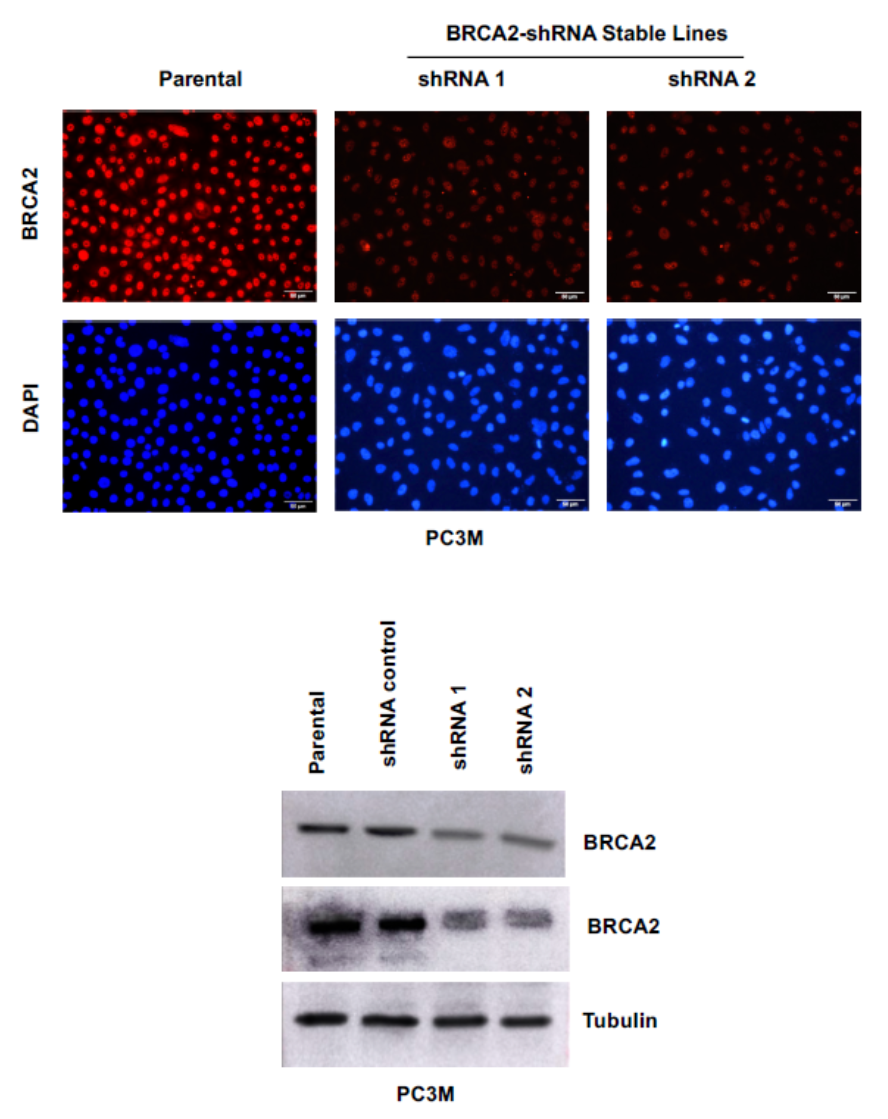


(D)

(C)

**Figure S3. Viability of parental or BRCA2 depleted PC3M cells in response to LP-184 or Olaparib.** PC3M parental and BRCA2 shRNA knockdown stable cell clones **(A)** treated with LP-184 for 3 days, **(B)** treated with Olaparib for 3 days, **(C)** treated with antiBRCA2 antibody and nuclear stain DAPI visualized by fluorescence microscopy and **(D)** immunoblotted for BRCA2
