# Supplementary material for: LP-184, a Novel Acylfulvene Molecule, Exhibits Anticancer Activity against Diverse Solid Tumors with Homologous Recombination Deficiency
Source: Cancer Res Commun. 2024 May 6;4(5):1199–210. doi: 10.1158/2767-9764.CRC-23-0554 (PMC11072798; doi:10.1158/2767-9764.CRC-23-0554)
Supplement: Supplementary Table S3 — Table S3 shows LP-184 IC50s in normal cells [file crc-23-0554-s06.docx]

**Supplementary Table S3.** **LP-184 IC50s in non-tumor normal epithelial cell lines.** LP-184 IC50s from 3-day treatment in two epithelial cell lines are listed.

| **Non-tumor normal epithelial cell line** | **LP-184 IC50 [nM]** |
| --- | --- |
| HPNE (pancreas) | 670 |
| PrEC (prostate) | 635 |
