# Supplementary material for: LP-184, a Novel Acylfulvene Molecule, Exhibits Anticancer Activity against Diverse Solid Tumors with Homologous Recombination Deficiency
Source: Cancer Res Commun. 2024 May 6;4(5):1199–210. doi: 10.1158/2767-9764.CRC-23-0554 (PMC11072798; doi:10.1158/2767-9764.CRC-23-0554)
Supplement: Supplementary Figure S4 — Figure S4 shows the in vivo tumor response in 8 TNBC PDX models to LP-184 single agent [file crc-23-0554-s07.docx]

**Supplementary Figure S4**.

(C)

(A)

(B)

**
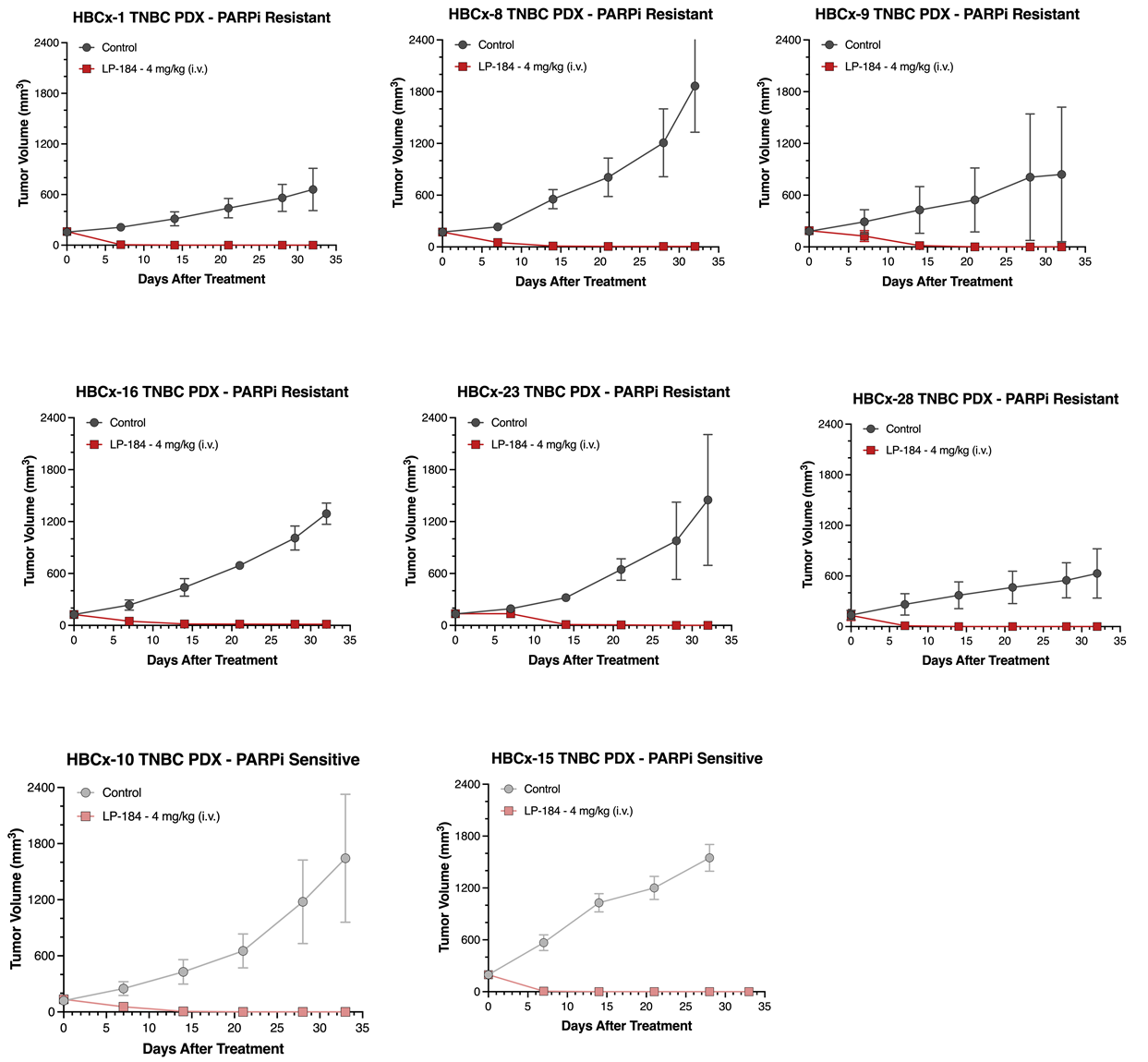
**

(F))

(E)

(D)

(H)

(G)

**Figure S4.** ***In vivo* anti-tumor efficacy of LP-184.** Tumor growth curves (mean +/- SD) across a range of HRD TNBC PDX models as shown in panels **(A)** – **(H)**, treated with 4 mg/kg i.v. LP-184 on Days 0, 2, 4, 6, 8 and 16, 18, 20, 22, 24.
