# Supplementary material for: LP-184, a Novel Acylfulvene Molecule, Exhibits Anticancer Activity against Diverse Solid Tumors with Homologous Recombination Deficiency
Source: Cancer Res Commun. 2024 May 6;4(5):1199–210. doi: 10.1158/2767-9764.CRC-23-0554 (PMC11072798; doi:10.1158/2767-9764.CRC-23-0554)
Supplement: Supplementary Figure S5 — Figure S5 shows the in vivo tumor response in a TNBC PDX model to LP-184 in combination with Olaparib [file crc-23-0554-s08.docx]

**Supplementary Figure S5**.


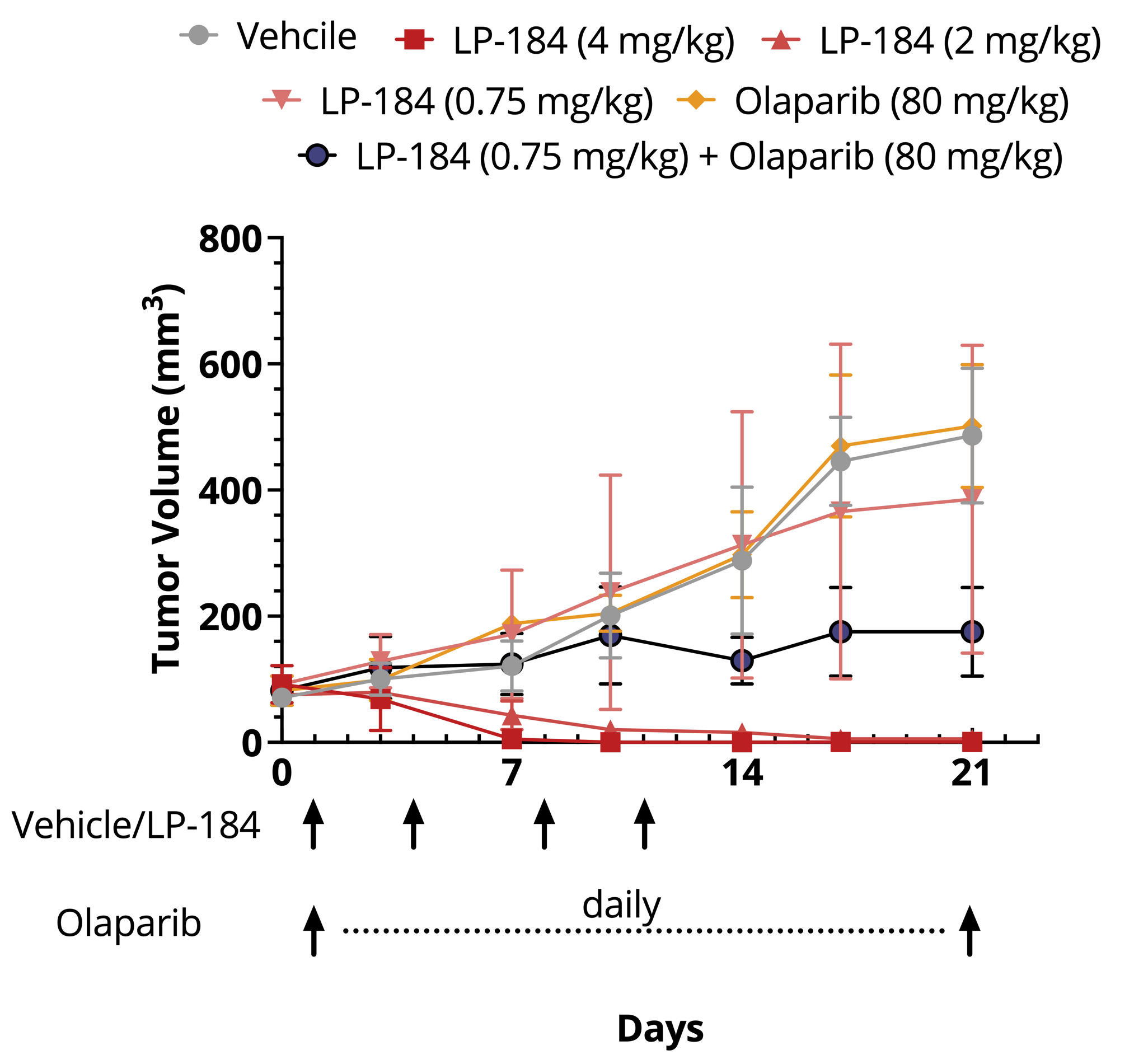

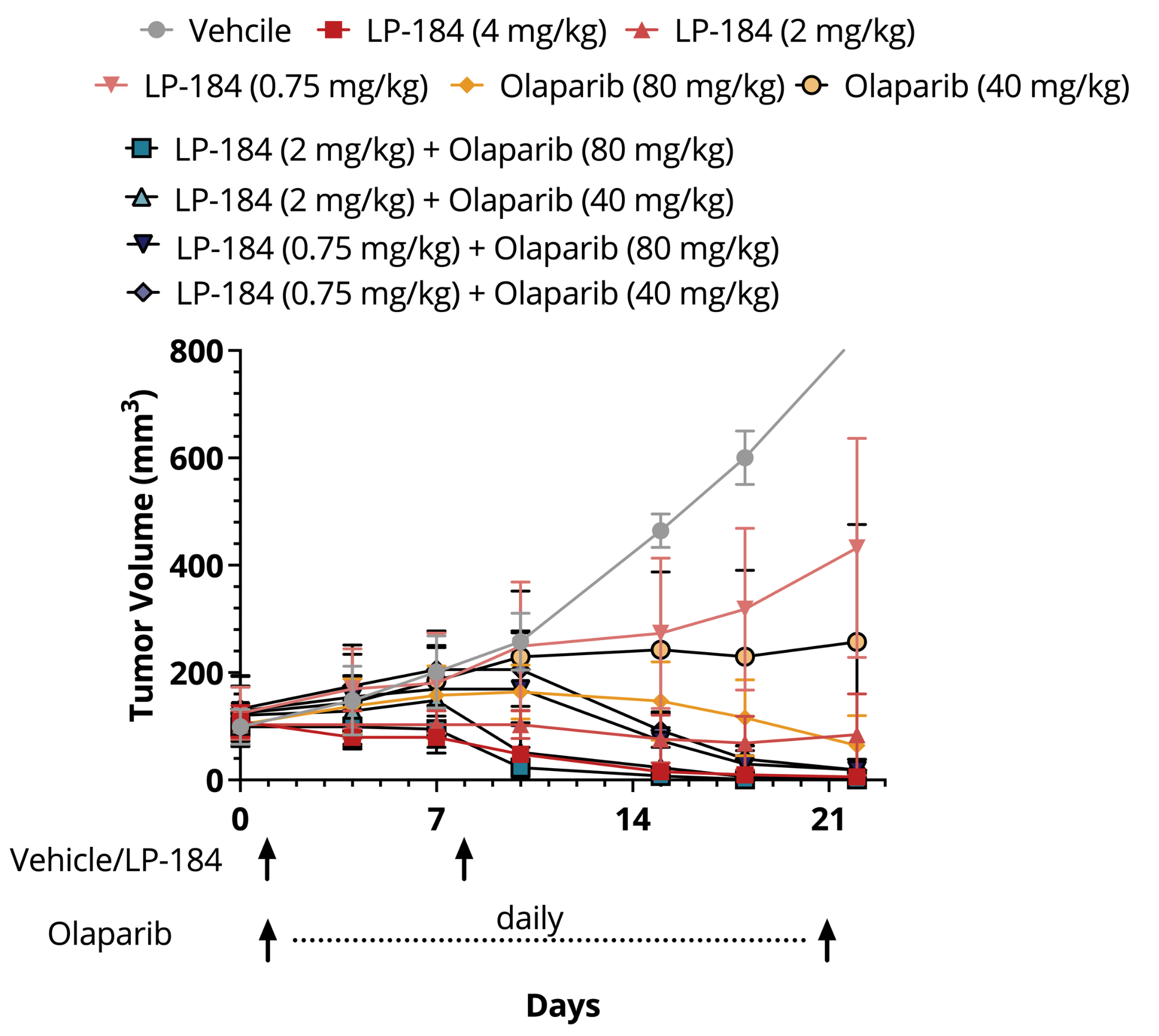


(B)

(A)

**Figure S5. *In vivo* anti-tumor efficacy of LP-184 in combination with Olaparib.** Tumor growth curves (mean +/- SD) in HBCx-10 **(A)** and HBCx-28 **(B)** TNBC PDX mouse models treated with vehicle, LP-184 (i.v.), olaparib (p.o.), or LP-184 plus olaparib. Dose schedules were indicated by arrows.
