# Supplementary material for: LP-184, a Novel Acylfulvene Molecule, Exhibits Anticancer Activity against Diverse Solid Tumors with Homologous Recombination Deficiency
Source: Cancer Res Commun. 2024 May 6;4(5):1199–210. doi: 10.1158/2767-9764.CRC-23-0554 (PMC11072798; doi:10.1158/2767-9764.CRC-23-0554)
Supplement: Supplementary Figure S6. — Figure S6 shows a heatmap with correlations between LP-184 activity in NCI0 cancer cell lines and transcript expression levels of Homoloous Recombination genes [file crc-23-0554-s09.docx]

**Supplementary Figure S6**.


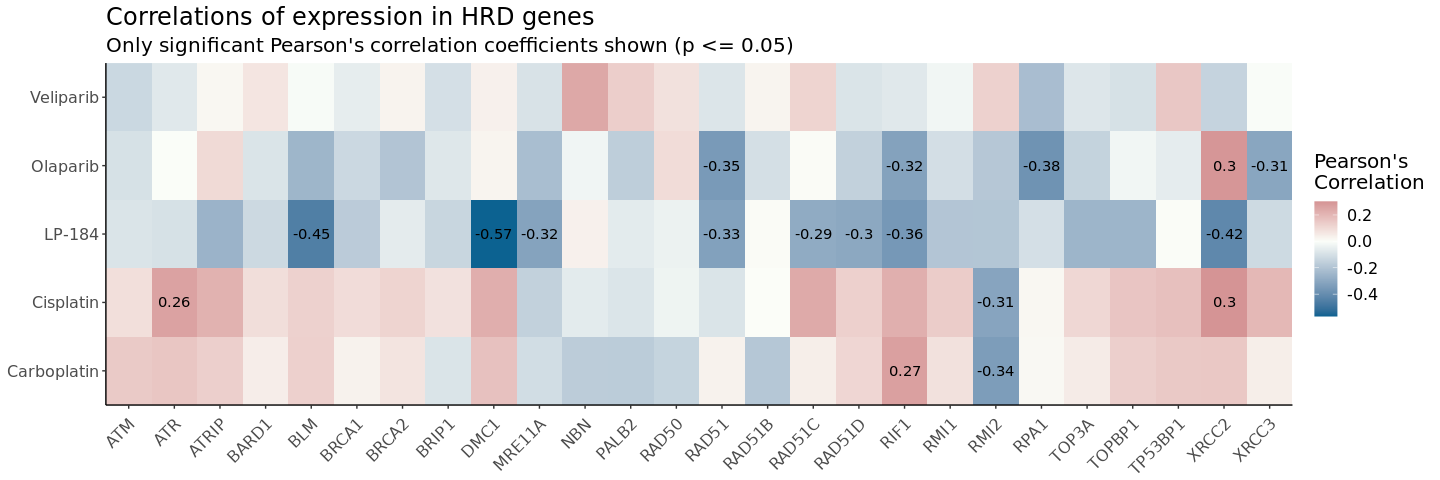


**Figure S6. LP-184 activity is negatively correlated with a panel of HR genes.** Correlation of 26 HR gene mRNA expression level with Veliparib, Olaparib, LP-184, Cisplatin and Carboplatin response in NCI60 cancer cell lines is plotted. Only the significant correlations (p value ≤ 0.05) are shown with correlation coefficients.
