# Supplementary material for: LP-184, a Novel Acylfulvene Molecule, Exhibits Anticancer Activity against Diverse Solid Tumors with Homologous Recombination Deficiency
Source: Cancer Res Commun. 2024 May 6;4(5):1199–210. doi: 10.1158/2767-9764.CRC-23-0554 (PMC11072798; doi:10.1158/2767-9764.CRC-23-0554)
Supplement: Supplementary Figure S7 — Figure S7 shows mouse body weight changes in selected TNBC PDX models following LP-184 treatment [file crc-23-0554-s10.docx]

**Supplementary Figure S7.**

**Figure S7. Mouse body weight changes as a measure of *in vivo* tolerability of LP-184.** Relative body weight change in TNBC PDX models HBCx-9, HBCx-10, HBCx-15, HBCx-23 and HBCx-28 treated with LP-184 or vehicle control.
