# Supplementary material for: LP-184, a Novel Acylfulvene Molecule, Exhibits Anticancer Activity against Diverse Solid Tumors with Homologous Recombination Deficiency
Source: Cancer Res Commun. 2024 May 6;4(5):1199–210. doi: 10.1158/2767-9764.CRC-23-0554 (PMC11072798; doi:10.1158/2767-9764.CRC-23-0554)
Supplement: Supplementary Figure S8 — Figure S8 shows mouse body weight changes in selected TNBC PDX models following LP-184 treatment [file crc-23-0554-s11.docx]

**Supplementary Figure S8**.

**Figure S8. Mouse body weight changes as a measure of *in vivo* tolerability of LP-184.** Relative body weight change in TNBC PDX models HBCx-1, HBCx-8, HBCx-16, HBCx-24 and T168 treated with LP-184 or vehicle control.
