# Supplementary material for: LP-184, a Novel Acylfulvene Molecule, Exhibits Anticancer Activity against Diverse Solid Tumors with Homologous Recombination Deficiency
Source: Cancer Res Commun. 2024 May 6;4(5):1199–210. doi: 10.1158/2767-9764.CRC-23-0554 (PMC11072798; doi:10.1158/2767-9764.CRC-23-0554)
Supplement: Supplementary Figure S10 — Figure S10 shows mouse body weight changes in a TNBC PDX model following treatment with LP-184, Olaparib or the combination [file crc-23-0554-s13.docx]

**Supplementary Figure S10**.

**Figure S10. Mouse body weight changes as a measure of *in vivo* tolerability of LP-184 combined with Olaparib.** Relative body weight change in the TNBC PDX model HBCx-28 treated with vehicle control, LP-184, Olaparib or LP-184 in combination with Olaparib.
