# Supplementary material for: LP-184, a Novel Acylfulvene Molecule, Exhibits Anticancer Activity against Diverse Solid Tumors with Homologous Recombination Deficiency
Source: Cancer Res Commun. 2024 May 6;4(5):1199–210. doi: 10.1158/2767-9764.CRC-23-0554 (PMC11072798; doi:10.1158/2767-9764.CRC-23-0554)
Supplement: Supplementary Figure S11 — Figures S11 shows LP-184 activity in normal cells [file crc-23-0554-s14.docx]

**Supplementary Figure S11**.

**Figure S11. *In vitro* activity of LP-184 in normal cells.** Dose response curves for LP-184 activity in non-tumor normal epithelial cell lines are shown. LP-184 in the concentration range of 1 nM – 10uM was tested in a 3-day MTT assay.
